# Entre a divulgação científica e a eugenia tardia: rupturas e permanências na trajetória intelectual de Salvador de Toledo Piza Jr., 1898-1988

**DOI:** 10.1590/S0104-59702023000100025

**Published:** 2023-07-10

**Authors:** Guilherme Prado Roitberg

**Affiliations:** i Programa de Pós-graduação em Educação Universidade Federal de São Carlos São Carlos SP Brasil guilhermeroitberg@gmail.com Doutorando, Programa de Pós-graduação em Educação/Universidade Federal de São Carlos. São Carlos – SP – Brasil guilhermeroitberg@gmail.com

**Keywords:** eugenia, Salvador de Toledo Piza Jr. (1898-1988, racismo científico, evolucionismo, história das ciências

## Abstract

O artigo analisa as rupturas e permanências do ideário eugênico na obra de Salvador de Toledo Piza Jr., professor e geneticista da Escola Superior de Agricultura “Luiz de Queiroz”. A partir da pesquisa documental sobre artigos, correspondências e anotações do ex-diretor do *Boletim de Eugenia* , investiga-se a reconfiguração da eugenia no contexto pós-1945, momento em que Piza Jr. passou a atuar como divulgador do evolucionismo. Conclui-se que Piza Jr. deixou de defender publicamente a eugenia na segunda metade do século XX, mas manteve a concepção racializada nos anos 1950, correspondeu-se com sociedades eugenistas nos anos 1960 e sustentou a interpretação hierarquizada de evolução humana até final dos anos 1980.

Ao analisar a reconfiguração da eugenia no decorrer da década de 1930, Stepan (2005) postulou que o racismo extremado nos EUA, França e principalmente Alemanha levou a críticas internas e externas, forçando a reavaliação da ciência do melhoramento racial em um plano mais amplo. As Leis de Nuremberg que proibiam os casamentos entre judeus e gentios e seu entrelaçamento com a legislação eugênica nazista começaram a ser mundialmente divulgadas e debatidas. As notícias chegavam à América Latina, mas a desaprovação da política reprodutiva nazista já era manifestada em linhas gerais na região. Na década de 1930, os EUA empreenderam uma espécie de reforma eugênica que cessou os investimentos nas instituições e reviu as posições racistas e classistas anteriores.

Stepan (2005) demonstrou que, em contrapartida, a eugenia ao final da década de 1930 enfraqueceu, mas, sem desaparecer, floresceu dos seus próprios escombros. Seu campo científico foi reconstituído como o novo campo da genética humana, um campo supostamente neutro e sem os fundamentos ideológicos da eugenia “clássica”. A eugenia se tornou, assim, menos “ideológica”, mais “científica”, liberal e progressista. Sua estrutura externa se modificou, mas seu núcleo estrutural que englobava questões de gênero, raça e classe se manteve. A eugenia seria, nessa perspectiva, uma ciência flexível, que conseguiu manter sob novos disfarces seu antigo compromisso com a manipulação do sexo e o controle hereditário. A reconfiguração via genética humana permitiu sua permanência, mesmo que a palavra “eugenia” continue até os dias atuais associada a uma má reputação ( Stepan, 2005 ).

Essa reconfiguração no contexto pós-Segunda Guerra Mundial (1939-1945) foi debatida por Carvalho e Souza (2017) , que demonstraram que a eugenia se manteve viva nos anos 1950 e 1960 a partir da atuação dos geneticistas, que intentaram desconstruir a imagem negativa da eugenia e desvinculá-la do racismo científico nazista. Sem pretender traçar uma história factual da eugenia desde sua estruturação científica com o polímata inglês sir Francis Galton (1822-1911) à sua reconfiguração, o presente trabalho parte do convite à investigação sobre as rupturas e continuidades na história da eugenia pós-1945 proposto por Carvalho e Souza (2017) . A partir de uma pesquisa documental sobre os artigos, palestras, ementas, correspondências e anotações de Salvador de Toledo Piza Jr. (1898-1988), professor e geneticista da Escola Superior de Agricultura “Luiz de Queiroz” (Esalq), analisamos tanto os silenciamentos quanto os possíveis “ecos” do ideário eugenista na obra do ex-diretor do *Boletim de Eugenia* .

Nossa pesquisa documental se concentra nas fontes encontradas na Biblioteca Salvador de Toledo Piza Júnior, no Protocolo, Seção de Expedientes, Assistência Administrativa e Financeira na Esalq em Piracicaba e no acervo do Centro de Documentação Histórica da Fundação Romi na cidade de Santa Bárbara d’Oeste. Conforme demonstraremos ao longo do artigo, essas duas cidades do interior paulista constituíram o epicentro da longa campanha de Piza Jr. pela disseminação dos conhecimentos evolucionistas, na posição de professor, pesquisador, divulgador científico, membro “ilustre” e frequentador ativo de clubes sociais, com destaque para as unidades barbarense e piracicabana do Rotary Club. Ademais, encontramos pequenas notas sobre Piza Jr. nos jornais disponíveis no Acervo Digital da Biblioteca Nacional, além dos artigos do professor esalquiano na *Revista de Agricultura* , da qual foi fundador, diretor e colaborador.

## Salvador de Toledo Piza Jr. e o movimento eugenista brasileiro

Nascido em Capivari (SP), Piza Jr. se formou em 1921 pela Esalq, ingressou no quadro de funcionários no ano seguinte como ajudante de gabinete interino da quinta cadeira (zootecnia) e, em 1931, tornou-se professor catedrático da nona cadeira (zoologia geral e especial, anatomia e fisiologia comparada dos animais domésticos). Desenvolveu toda sua carreira como docente e pesquisador na Esalq, da graduação à aposentadoria. Ao lado de Octavio Domingues (1897-1972), professor, geneticista e colega de trabalho na faculdade piracicabana, Piza Jr. dirigiu e editou, entre 1932 e 1933, o *Boletim de Eugenia* .^1^

Piza Jr. foi um intelectual mais “teórico” nas discussões sobre genética mendeliana em comparação a Kehl e Domingues. Aproximando-se da tradição de pesquisa alemã, país no qual estudou biologia e zoologia nas escolas superiores de agricultura e veterinária de Berlim, abarcou em sua trajetória acadêmica diversas áreas de interesse, com especial dedicação aos temas da evolução e hereditariedade. Acadêmico renomado e reconhecido por sua dedicação à ciência e à Esalq, Piza Jr. destacou-se no campo da entomologia, mas escreveu sobre temas variados no campo da biologia, genética, agricultura, política, teologia e eugenia. Publicou tanto em jornais de grande circulação, como *O Estado de S. Paulo* e *Folha da Manhã* , como em periódicos de menor circulação, como o *Jornal de Piracicaba* e o *Diário de Piracicaba* ( Habib, 2010 ).

O primeiro registro da campanha eugênica de Piza Jr. consiste no artigo “Anotações à margem das ‘Lições de eugenia’ do Dr. Renato Kehl”, publicado em 1930, na *Revista de Agricultura* . Piza Jr. (1930) elogiou e apontou correções na obra de Renato Kehl, que as inseriu na segunda edição de *Lições de eugenia* , publicada em 1935 (cf. Kehl, 1935 ). A partir da segunda fase do *Boletim de Eugenia* , dirigido e editado em Piracicaba, Piza Jr. passou a assinar seus textos como membro da Comissão Central Brasileira de Eugenia (CCBE),^2^ publicando artigos diretamente relacionados ao tema, nos quais defendeu as teses radicais do racismo científico. Destacamos os textos “O que pode resultar do casamento entre o branco e o preto” (Piza Jr., jan.-mar. 1932), “O casamento do branco com o preto à luz da biologia” (Piza Jr., abr.-jun. 1932) e “A hereditariedade da cor da pele no casamento branco-preto” (Piza Jr., jul.-set. 1932, jan.-mar. 1933).

Os artigos de Piza Jr. (jan.-mar. 1932, abr.-jun. 1932, jul.-set. 1932, jan.-mar. 1933, abr.-jun. 1933) publicados no *Boletim de Eugenia* se estruturam na genética mendeliana para a explicação dos fenômenos da hereditariedade. A análise pormenorizada de seus textos nesse periódico nos permite afirmar que a figura de Mendel foi mais preponderante em sua concepção eugênica do que propriamente a de Galton, o que não significa que Piza Jr. ignorasse as contribuições e o pioneirismo do polímata britânico na estruturação científica da eugenia, conforme exposto no artigo “Um programa para a eugenia”, publicado na última edição do *Boletim de Eugenia* , em abril-junho de 1933 (Piza Jr., abr.-jun. 1933). A eugenia de Piza Jr. (abr.-jun. 1932) também se orientava a partir de outros autores baseados em Mendel, como o geneticista norte-americano e pioneiro nos estudos sobre a mosca drosófila William Ernest Castle (1867-1962), o naturalista suíço Arnold Lang (1855-1914) e o geneticista sueco Nils Herman Nilsson-Ehle (1873-1949).

Ao lado de uma gravura de Piza Jr. e de seu título de doutor *honoris causa* concedido pelo Instituto de Zoologia de Berlim (1923), a Biblioteca Salvador de Toledo Piza Júnior, na Esalq, possui três quadros emoldurados, que denotam os principais referenciais teóricos do professor: o biólogo austríaco Gregor (Johann) Mendel (1822-1884), o biólogo e naturalista alemão Ernst (Heinrich Philipp August) Haeckel (1834-1919) e o biólogo e naturalista britânico Charles (Robert) Darwin (1809-1882). Conjuntamente à ampla coleção de livros, muitos dos quais escritos pelo esalquiano no decorrer de sua carreira como professor e geneticista da Esalq, seu acervo possui um catálogo com mais de mil trabalhos publicados em seu nome. Somente na *Revista de Agricultura* foram registrados 222 textos de sua autoria, publicados entre 1926, ano de fundação do periódico piracicabano, e 1987, poucos meses antes de seu falecimento. Isso posto, consideramos preliminarmente não ser possível resumir a esses três autores os fundamentos epistemológicos da ciência produzida por um intelectual que atuou em diversos campos – da teologia à zoologia – ao longo de seus 89 anos de vida.

Embora também adepto do racismo científico compartilhado por Kehl (1935) e discordando da viabilidade da mestiçagem defendida por Domingues (1936) , a concepção racial de Piza Jr. era original e distinta das de seus colegas de *Boletim de Eugenia* . Para Piza Jr. (jan.-mar. 1933, p.8-10), era possível a existência de indivíduos “perfeitamente brancos”, assim como indivíduos “perfeitamente pretos”. Essa passagem é fundamental, pois denota que a eugenia do professor esalquiano não se baseava na ideia de que os negros “puros” eram sinônimo de degeneração, uma vez que esta somente ocorria a partir da mistura entre a “espécie” branca e a “espécie” negra. Dito de outro modo, Piza Jr. (jan.-mar. 1933) considerava que um negro “puro” poderia ser um indivíduo perfeito do ponto de vista eugênico, assim como um indivíduo branco “puro”.

O racismo científico de Piza Jr. (jan.-mar. 1933) se alicerçava na compreensão de que brancos e negros pertenciam a “espécies” distintas. Esse entendimento rechaçava a miscigenação, pois essa gerava indivíduos “mulatos”, “híbridos” e “inferiores” por advirem de uniões interespecíficas. Para o professor, o “instinto de aversão” era uma característica comum entre todos os animais. Essa “repulsa” garantia que espécies distintas não se reproduzissem, mas se vissem como concorrentes na luta pela sobrevivência. O homem teria o poder de dominar seus instintos por meio da inteligência, mas esse domínio era limitado. Para Piza Junior (jan.-mar. 1933), essa “aversão” biológica existia no inconsciente dos homens, apesar de geralmente estar adormecida. Não existiria qualquer atração espontânea, afinidade histórica ou aproximação filogenética entre as “espécies” branca e negra. Segundo Piza Jr. (jan.-mar. 1933), tanto do ponto de vista poligênico quanto do ponto de vista monogênico, a união entre o branco e o negro era biologicamente “antinatural” e socialmente “repugnante”, motivo pelo qual deveria ser evitada.

De acordo com Habib (2010) , Piza Jr. publicou no *Jornal de Agronomia* da Esalq, em 1938, o artigo “Em torno da antropologia”, no qual propôs que o estudo da espécie humana deveria seguir as mesmas metodologias e regras do estudo zoológico de cavalos e macacos, aplicando a mesma divisão entre espécies da zoologia aos seres humanos. Essa proposta se embasava na crença de Piza Jr. de que dentro da divisão *Homo sapiens* coexistiam “espécies” diferentes, como a branca e a negra. Habib (2010) ressaltou que, apesar de o artigo publicado em 1938 não possuir aparente relação com eugenia, ele explicitou as ideias defendidas por Piza Jr. nos anos 1920 e 1930. A principal delas foi a tese da “aversão” e atração entre as “espécies” delineada na conclusão do artigo “A hereditariedade da cor da pele no casamento branco-preto”, publicado na penúltima edição do *Boletim de Eugenia* (Piza Jr., jan.-mar. 1933).

Segundo Habib (2010 , p.276-277), o esalquiano considerou que “o princípio da aversão seria um dos principais fatores da evolução” e criticou o conceito de “espécie”, que não explicava de forma satisfatória a evolução humana em suas várias novas “subespécies” ou “ramificações”. Considerando a união interracial um comportamento irracional e irrefletido, o professor postulou que o cruzamento interespecífico entre brancos e negros estava no mesmo patamar de desrazão e anormalidade que a zoofilia, definindo a coexistência entre as duas “espécies” como uma coabitação psicótica. Para o professor, adeptos a um princípio de fraternidade humana sem fundamento científico, os intelectuais não compreendiam o problema das raças, resistindo à subdivisão do gênero em “espécies”, negando, assim, a verdade biológica ( Habib, 2010 ).

A ideia apresentada no artigo “Em torno da Antropologia” a partir do conceito de “instinto de aversão” representou uma continuidade ou complementação do conceito de “repulsa biológica” desenvolvido no artigo “A hereditariedade da cor da pele no casamento branco-preto” (jan.-mar. 1933), argumento central na concepção eugênica de Piza Jr. diagnosticado nos trabalhos de Habib (2010) e Wegner (2017) . A artigo também denotou que Piza Jr. manteve pelo menos até o final da década de 1930 mais continuidades do que rupturas no tocante à sua leitura racista e contrária à miscigenação. Vejamos agora, a partir de outros documentos, indícios de permanências e rupturas ao longo da trajetória intelectual do professor da Esalq na segunda metade do século XX.

## A campanha evolucionista de Piza Jr. no contexto pós-1945

Em 1944, o jornal *Cidade de Santa Bárbara* publicou uma nota informando sobre a visita dos membros do Rotary Club de Piracicaba à Santa Bárbara d’Oeste para uma reunião-jantar no Clube Barbarense. O jantar também contou com a presença de uma caravana de rotarianos de Americana, cidade vizinha de Santa Bárbara d’Oeste. A nota informou que discursou nessa reunião o presidente do Rotary Club de Piracicaba, doutor Salvador de Toledo Piza Jr., que agradeceu a calorosa recepção dos seus coirmãos barbarenses e afirmou que aquele era o início de uma cordial relação entre as cidades vizinhas e amigas. O texto não apresentou nenhuma menção à eugenia, mas foi o primeiro a revelar a influência do professor esalquiano sobre os clubes sociais da região e sua posição de prestígio como professor da Universidade de São Paulo e presidente dos rotarianos de Piracicaba (Rotary Club, 17 dez. 1944).

Em 28 janeiro de 1945, o jornal *Cidade de Santa Bárbara* informou que o “ilustre” professor Piza Jr. esteve presente na entrega da Carta Constitucional ao Rotary Club Local (A entrega..., 28 jan. 1945). Em setembro do mesmo ano, o jornal publicou uma nota informativa sobre uma reunião realizada no Rotary Club de Santa Bárbara d’Oeste na qual foi discutido o “problema da Educação”. A reunião contou com a presença de diversos educadores e empreendedores paulistas e mineiros, dentre os quais o industrial Américo Emílio Romi (1896-1959) e Alfredo Maluf (1903-1951), industrial e ex-prefeito da cidade de Santo André (SP). Entre os nomes dos palestrantes da reunião, consta o do professor Salvador de Toledo Piza Jr. (Uma brilhante..., 2 set. 1945). Não encontramos informações sobre qual “problema da Educação” a palestra versava.

Em 1949, o nome do professor Piza Jr. apareceu na coluna de notícias do periódico barbarense *Jornal D’Oeste* . Em nota, o jornal informou que o professor esalquiano estava entre os ilustres convidados de uma reunião-jantar no Rotary Club local, e que sua palestra, “Inteligência dos animais”, foi aclamada entre os rotarianos (Rotary Club, 17 jul. 1949). Em 14 de dezembro de 1952, o periódico barbarense *O Jornal do Povo* publicou uma nota mencionando que no dia 16 daquele mês, às 20 horas, o professor catedrático da Esalq realizaria “uma importante conferência literocientífica abordando interessante tema”, sem mencionar, todavia, qual seria o assunto tratado (Notícias Locais, 14 dez. 1952, p.1). Essas duas notas explicitam que, no final da década de 1940 e início dos anos 1950, Piza Jr. continuava palestrando sobre temas relacionados à biologia, mantendo seu compromisso com a divulgação científica entre o público leigo.

Identificamos na Biblioteca Salvador de Toledo Piza Júnior uma correspondência enviada a Piza Jr. pelo biólogo e geneticista Ademar Freire-Maia (12 out. 1952). Na carta datilografada em papel timbrado da Faculdade de Filosofia da Universidade do Paraná e datada de 12 de outubro de 1952, Freire-Maia informou que o Centro de Estudos de História Natural de sua instituição realizara há poucos dias a Semana Evolucionista, na qual diversos cientistas discutiram temas relacionados à origem das espécies. Dado o sucesso do evento, novas conferências seriam realizadas, para as quais Piza Jr. estava convidado a participar como conferencista. Freire-Maia (12 out. 1952) justificou o convite elogiando a “cultura invulgar” da qual Piza Jr. era possuidor e seu interesse permanente pelas questões da ciência, da qual era “especialista brilhante”.

Em 20 de outubro de 1957, Piza Jr. enviou uma carta datilografada a Alberto Amaral, agradecendo uma carta recebida no dia 1° de setembro. Piza Jr. (20 out. 1957) explicitou nessa correspondência que compreendia a ciência como um processo contínuo de questionamentos e descobertas, expondo seu “espírito filosófico” criticado pelo geneticista alemão e colega de Esalq Friedrich Gustav Brieger (1900-1985)^3^ ( Habib, 2010 ). O professor explanou que continuava interessado no tema da evolução humana, carro-chefe de suas preocupações no campo da filosofia da ciência. A carta de Piza Jr. (20 out. 1957) se encerrou mandando abraços ao seu amigo Amaral, trazendo provocações com relação ao tema da hereditariedade, sem mencionar, contudo, as teses da eugenia. Em contrapartida, a análise minuciosa sobre as publicações de Piza Jr. pós-1945 revela que, mesmo sem abordar especificamente o tema da ciência do melhoramento racial, o esalquiano não renunciou à interpretação racializada sobre a população brasileira.

Em junho de 1958, a *Revista de Agricultura* publicou o texto “A participação do índio, do branco e do negro na etnia brasiliana”, transcrição do discurso proferido por Piza Jr. na sessão de colação de grau da turma dos engenheiros-agrônomos da Esalq, realizada em 8 de março de 1957 no salão nobre da instituição piracicabana. No tocante a esse evento, o professor havia sido escolhido para proferir o discurso aos formandos. Piza Jr. (jun. 1958) iniciou sua fala homenageando os alunos, parabenizando-os por sua relação de gratidão tanto com o corpo docente quanto com a instituição. Agradeceu o convite e afirmou que contaria uma pequena história a partir de seu “lirismo inato”, que nem mesmo as ciências “duras” foram capazes de sufocar. Curiosamente, o tema da curta história escolhida por Piza Jr. (jun. 1958) foi a participação do “índio”, do branco e do negro na constituição étnica do país. O texto não informou os motivos que levaram o professor a inserir um assunto dessa natureza no discurso de formatura de uma turma de engenharia agronômica.

Em seu discurso, Piza Jr. (jun. 1958, p.68) explicou a formação étnica brasileira, descrevendo o “índio” como “guerreiro”, o branco como “invasor” e denunciando a violência e a escravidão contra a população nativa. Na sequência, o professor expôs que, no final dos anos 1950, mesmo sem mencionar a questão da degeneração via mestiçagem, ainda acreditava na divisão racializada da população brasileira que defendera em seus artigos ao longo dos anos 1930. Em uma interpretação reducionista e caricatural, Piza Jr. (jun. 1958, p.68) considerou o miscigenado um produto híbrido que, não sendo nem branco nem indígena, herdou as características de cada uma dessas distintas “raças”: “Do abraço amoroso, sob o céu tropical, nasceu uma gente que índia não era, e sem ser portuguesa, tinha do luso a força e a destreza; dos filhos da selva, o amor e a pureza” (Piza Jr., jun. 1958, p.68).

O professor descreveu a conversão do indígena ao cristianismo a partir da ação dos jesuítas e, na sequência, a chegada do negro, retratado como um elemento trabalhador e honrado, apesar de escravizado e violentado. Na parte final de seu discurso, Piza Jr. (jun. 1958) retratou de forma “poética” uma negra que “se entregou” sexualmente ao seu “sinhô” em troca de presentes; uma forma “descontraída” de suavizar e inserir em um discurso de formatura de uma das principais universidades do país a violência sexual cometida contra as mulheres negras. O estupro e a violência do sistema escravista se tornaram, no discurso de Piza Jr. (jun. 1958), uma corriqueira e caricatural “troca de favores”. Abaixo, a última frase do discurso transcrito na *Revista de Agricultura* :

E desse amor clandestino, que Deus sei lá se abençoou, nasce um ente misterioso, feito de branco e de preto, e que sem ser preto nem branco era branco e era preto: o mulato, cor trigueira, veio a todos demonstrar, que na terra brasileira duas raças se juntaram para não mais separar (Piza Jr., jun. 1958, p.68).

O texto se encerrou nesse trecho, aparentemente inacabado. Na página seguinte dessa edição da *Revista de Agricultura* se inicia um texto de outro autor. Teria Piza Jr. (jun. 1958) encerrado dessa forma seu discurso aos alunos em pleno salão nobre da Esalq? Em caso negativo, qual teria sido o desfecho dessa fala e por que ela não foi transcrita? Que motivos levaram Piza Jr., na posição de diretor da *Revista de Agricultura* , a inserir esse discurso nas páginas de um periódico científico? O discurso demonstrou que Piza Jr. (jun. 1958) manteve sua crença no “mulato” como um híbrido formado a partir da mistura entre duas raças “puras”, de origens distintas. Indagamos, finalmente, se em 1958, cerca de duas décadas após a publicação dos textos mais racistas de Piza Jr., o professor ainda considerava o mestiço como um “degenerado” e o casamento do negro com o branco como uma união interespecífica “abominável”, “repugnante” e irracional a ponto de ser equiparada à zoofilia.

Meses após o “intrigante” discurso de formatura, Piza Jr. continuou suas atividades como divulgador científico, proferindo um discurso intitulado “A vida é uma luta: viver é lutar”, no Rotary Club de Santa Bárbara d’Oeste no dia 1º de julho de 1958 (Recordando, 13 jul. 2017). O conteúdo do discurso nos é desconhecido. Entre 1959 e 1960, Piza Jr. analisou na *Revista de Agricultura* o livro *Evolução* (publicado em 1941), escrito pelo padre Alejandro Roldán, professor de antropologia e psicologia na Faculdade de Filosofia da Companhia de Jesus na Espanha, com tradução para o português, de 1958, e notas elaboradas pelo padre Emanuel C. Rondon do Amarante, professor de biologia na Pontifícia Faculdade de Filosofia da Companhia de Jesus no Brasil. A série de cinco artigos intitulada “‘Evolução’, do padre Roldán, S.J.” é primordial para compreender os fundamentos epistemológicos do evolucionismo de Piza Jr., bem como a maneira particular como o esalquiano buscou associar a teologia cristã ao darwinismo, elemento que marcou suas atividades como divulgador científico e esteve presente em suas publicações até o final dos anos 1980 (Piza Jr., mar. 1959, jun. 1959, set. 1959, dez. 1959, mar. 1960).^4^

O professor elaborou uma resenha crítica, discordando em diversos pontos tanto do autor quanto das notas do tradutor, mas recomendando a obra por constituir um indicativo de que a Igreja católica aceitou a evolução como uma verdade científica. Piza Jr. (jun. 1959) afirmou que, apesar dos teólogos católicos serem apenas parcialmente evolucionistas, a aceitação dos fatos cientificamente comprovados da evolução representava um enorme progresso. Ao longo de sua análise, Piza Jr. (mar. 1959) explicitou sua inspiração em Haeckel, considerando-o o cientista que mais se esforçou na vulgarização do evolucionismo de Darwin, publicando obras acessíveis ao público leigo e desconstruindo preconceitos historicamente promulgados pelo fundamentalismo religioso. De acordo com Piza Jr. (mar. 1959), o principal erro do cristianismo foi ter combatido as teses evolucionistas sem compreender que a evolução ocorreu pela vontade expressa de Deus.

Segundo Piza Jr. (mar. 1959), a Igreja católica deveria ter acatado a perspectiva do filósofo Santo Agostinho (354-430), segundo o qual Deus criou um mundo material que evoluiu de acordo com regras de sua própria autoria, ou seja, as leis que regem a transformação de todos os corpos do universo estavam contidas no ato da Criação. O que Deus criou, conforme Piza Jr. (jun. 1959, p.82), não foram espécies, mas indivíduos que se uniram sexualmente para povoar a Terra, que só então deram origem às espécies zoológicas: “Somente depois de Lamarck e Darwin é que se ficou sabendo que no pensamento de Deus achava-se condensado todo o processo de evolução pelo qual se formaram e continuaram a se formar as espécies que a Sistemática Zoológica vai cada vez mais catalogando”.

Piza Jr. (jun. 1959) criticou os criacionistas que, por colocarem o dogma acima da ciência, consideravam a evolução um pecado e as origens primatas do homem uma heresia. Concatenando teologia cristã e darwinismo, o professor defendeu um “cristianismo esclarecido” que, sem renunciar às suas tradições, deveria aceitar integralmente a evolução como um fato, substituindo a interpretação literal de “criação” pela concepção biológica de “transformação”. Piza Jr. (jun. 1959) foi enfático ao afirmar que não existia lugar para criacionismo e geração espontânea no campo da biologia, explicando que, em vez da criação espontânea delineada no Gênesis, o que comprovadamente ocorreu foi a transformação de seres microscópicos nas mais diversas espécies. Outrossim, Deus não teria criado as espécies em sua forma final, mas sim as condições para que elas evoluíssem a partir de um processo lento e progressivo de transformações.

Para Piza Jr. (mar. 1960), após Darwin, a evolução deixou de ser um problema para a ciência, tornando-se um fato histórico e uma verdade científica, sendo um impasse apenas para os religiosos não esclarecidos. A partir do arquétipo do padre Roldán, o esalquiano buscou demonstrar aos leitores da *Revista de Agricultura* que era cientificamente possível conciliar as teses da evolução com o cristianismo que não se fechava para os avanços da ciência e da tecnologia (Piza Jr., set. 1959). O professor encerrou sua apreciação sobre o livro *Evolução* enaltecendo o centenário da *Origem das espécies* (1859), de Darwin, e minimizando as críticas dos criacionistas a Haeckel por ter falsificado imagens de embriões para comprovar as teses evolucionistas em 1868,^5^ considerando que o erro do biólogo alemão em nada diminuía o valor científico de sua obra e do darwinismo (Piza Jr., mar. 1960).

No dia 13 de julho de 1961, o aluno Murilo Graner enviou a Piza Jr. uma correspondência em papel timbrado do Centro Acadêmico Luiz de Queiroz, organizado pelos discentes do curso de agronomia. Assinando como diretor do departamento científico, Graner (13 jul. 1961) informou ao professor que os departamentos Científico, Cultural e Artístico do Centro Acadêmico pretendiam realizar no segundo semestre um ciclo de palestras culturais e científicas, indagando se Piza Jr., na posição de criador de uma “revolucionária” teoria cromossômica,^6^ poderia realizar em agosto ou setembro um curso sobre evolução e genética. A carta de Graner (13 jul. 1961) denotou que, mesmo marginalizado no campo da genética,^7^ Piza Jr. era considerado por seus alunos como uma referência no assunto.

Localizamos documentos na Biblioteca Salvador de Toledo Piza Júnior que revelam que, se por um lado o estilo “polêmico” levou à marginalização de Piza Jr. entre os geneticistas, por outro, contribuiu para projetá-lo como divulgador científico entre a comunidade leiga. O professor guardou em seu acervo um recorte do jornal *Folha de Piracicaba* de 4 de agosto de 1961 cujo título era “Detentor do Prêmio Nobel de Medicina será refutado pelo Prof. Toledo Piza”. A matéria informou que Piza Jr. participou da 13ª Reunião da Sociedade Brasileira para o Progresso da Ciência realizada em Poços de Caldas (MG), na qual apresentou o trabalho “Evolução do conceito de gen”. Informou também que Piza Jr. promoveria no mês de agosto uma palestra com o tema “Evolução da espécie”, na qual discutiria e refutaria a opinião de Hermann Joseph Muller (1890-1967), geneticista norte-americano e vencedor do prêmio Nobel de Medicina (Detentor..., 4 ago. 1961).

Em 24 de agosto de 1961, o *Jornal de Piracicaba* anunciou o Curso de Evolução que Piza Jr. ministraria na Faculdade de Farmácia e Odontologia de Piracicaba a partir do dia 29. Os promotores do curso eram o Centro Acadêmico Luiz de Queiroz e o Centro Acadêmico XXI de Abril da Faculdade de Farmácia e Odontologia de Piracicaba. O jornal descreveu o professor como um cientista renomado internacionalmente, brilhante conferencista e defensor da teoria do cromossomo-unidade na transmissão dos caracteres hereditários, descrita como a “teoria que nega a existência do gen, corpúsculo que seria responsável pela determinação e transmissão de um ou mais caracteres, e que estaria localizada no cromossomio”^8^ (Na Faculdade..., 24 ago. 1961, s.p.).

Um dos últimos itens do programa do Curso de Evolução de Piza Jr., denominado “instinto e inteligência” (Na Faculdade..., 24 ago. 1961), pode constituir um indicativo de que o professor manteve, mesmo após o cessar de suas publicações explícitas sobre a eugenia e escamoteada nas entrelinhas de suas atividades como divulgador do evolucionismo, sua concepção pautada no controle dos instintos sexuais “animalescos” por intermédio da consciência eugênica. Essa possível permanência foi exposta no documento intitulado “Temas do II Curso de Evolução” (Piza Jr., 1961), o qual encontramos na Biblioteca Salvador de Toledo Piza Júnior. Trata-se do programa do curso ministrado por Piza Jr. na Faculdade de Odontologia de Piracicaba durante os meses de setembro a outubro de 1961.

O documento foi datilografado em duas páginas. Na primeira, consta na íntegra o programa do curso; na segunda, abaixo da palavra “aprende” grafada em caixa-alta, Piza Jr. (1961) redigiu as máximas a ser ensinadas ou os objetivos a alcançar ao longo do curso. Após as informações iniciais, Piza Jr. (1961) apresentou em tópicos o programa e, na página 2, após a palavra “aprende”, registrou as quatro lições principais de seu Curso de Evolução. Primeiramente, a ideia de evolução como esclarecimento dos enigmas do universo e da vida; em segundo lugar, a evolução como caminho para encontrar Deus; em terceiro lugar, a concepção de evolução como a criação do corpo e da alma, ambas dependentes do corpo animal, sendo o espírito subordinado à natureza biológica; finalmente, a necessidade do autoconhecimento mediante o estudo da evolução, um “Conhece-te a ti mesmo” a partir das bases evolutivas (Piza Jr., 1961).

O esalquiano delineou os objetivos pedagógicos do Curso de Evolução em cinco pontos que, conjuntamente, denunciam uma possível manutenção da concepção eugênica apresentada no artigo “A hereditariedade da cor da pele no casamento branco-preto (conclusão)”, de 1933, e indiretamente no artigo “Em torno da antropologia”, de 1938. Para Piza Jr. (1961), o homem não poderia esquecer suas origens evolutivas e não poderia ser um macaco, ou seja, precisava se portar como a espécie que ocupa o topo da escala evolutiva, orientando suas ações por meio da razão. Esses dois primeiros ensinamentos eram aprofundados na terceira orientação, segundo a qual o homem, como detentor da razão, não poderia deixar que o comportamento instintivo do macaco o dominasse. No penúltimo item, Piza Jr. (1961) postulou que o homem deveria dominar suas forças sexuais e interiorizar seus “instintos bestiais” quando em contato com a mulher, educando-se pela evolução, grafada com “E” maiúsculo, que o guiaria pelo caminho da verdade.

Não pretendemos que nossa hipótese recaia em uma análise a-histórica, afirmando que as ideias do professor esalquiano permaneceram inalteradas 31 anos após a publicação de “Anotações à margem das ‘Lições de eugenia’ do Dr. Renato Kehl”, na *Revista de Agricultura* , seu primeiro artigo sobre eugenia e a primeira contribuição de Piza Jr. (1930) para a campanha eugênica no Brasil. Qualquer análise que sugira uma continuidade sem rupturas, ignorando os diferentes contextos nos quais os documentos foram produzidos, poderia pecar pela imprecisão e pela generalização. Entretanto, mesmo não defendendo explicitamente a eugenia, o programa do Curso de Evolução nos permite afirmar que o controle do sexo pela razão, que alicerçou a concepção eugênica de Piza Jr., permaneceu vivo, mesmo que implicitamente, em sua trajetória intelectual.

As atividades de Piza Jr. como divulgador do evolucionismo continuaram ao longo da década de 1960 por meio de palestras nos clubes sociais e instituições de ensino no interior paulista. Em 13 de outubro de 1963, um ano após Piza Jr. receber o título de Cidadão Piracicabano (Município..., 7 set. 1962), o periódico barbarense *Jornal d’Oeste* publicou um texto sobre as festas do Jubileu de Ouro do Grupo Escolar José Gabriel de Oliveira, descrevendo a programação do evento que contou com atividades diversas para crianças, pais e educadores, com destaque para uma palestra de Piza Jr., em 25 de outubro, intitulada “Acerca da conduta animal” (Grupo Escolar..., 13 out. 1963). Essa mesma palestra foi mencionada na coluna “Recordando” do *Diário de Santa Bárbara d’Oeste* nos dias 3 de novembro de 2018 e 22 de novembro de 2019 (Recordando, 3 nov. 2018, 22 nov. 2019).

## A Sociedad Mexicana de Eugenesia e a reconfiguração “humanista” da eugenia

Se as palestras nos clubes sociais, instituições de ensino e as publicações de Piza Jr. na *Revista de Agricultura* não nos permitem afirmar com precisão que o professor continuou defendendo as teses racistas e radicais da eugenia como o fez em seus artigos na década de 1930, não nos restam dúvidas no que tange ao envolvimento do professor com intelectuais e instituições eugenistas internacionais ao longo da década de 1960. A primeira correspondência que identificamos na Biblioteca Salvador de Toledo Piza Júnior, enviada em nome da Sociedad Mexicana de Eugenesia,^9^ apresenta envelope carimbado com a data de 11 de outubro de 1960. Datilografada e assinada por Miguel López Esnaurrízar (presidente) e Alfredo M. Saavedra (M.) (secretário), a carta aparenta ser a primeira enviada pelos eugenistas mexicanos a Piza Jr., escrita em linguagem formal, apresentando ao colega brasileiro os anseios e objetivos da Sociedad Mexicana de Eugenesia (Esnaurrízar, Saavedra, 11 out. 1960).

Esnaurrízar e Saavedra (11 out. 1960, p.1) afirmaram que o lema da Sociedad Mexicana de Eugenesia era “ *Por una humanidad mejor* ”, frase que se repete em outras correspondências, sempre grafada em caixa alta. Segundo os autores, captando a essência das correntes filosóficas hodiernas, a sociedade se orientava pelo “humanismo”, em oposição à concepção dominante entre as instituições científicas que não destacavam as características “tipicamente humanas” da eugenia. Consideraram que o homem deveria ser distinguido do reino animal, em uma posição de destaque condizente com a dignidade humana. Na sequência, os mexicanos pediram a colaboração do eugenista brasileiro na promoção dessa concepção “humanista” de eugenia, segundo a qual a expressão “espécie humana” deveria desaparecer por pertencer ao campo da zoologia (Esnaurrízar, Saavredra, 11 out. 1960).

Os eugenistas mexicanos sugeriram que essa nova concepção de eugenia fosse disseminada por meio das organizações científicas e da educação, englobando da literatura pedagógica especializada às instituições universitárias. Somente assim, afirmaram Esnaurrízar e Saavedra (11 out. 1960), o homem poderia se aproximar do seu mais elevado ideal. Os eugenistas se colocaram à disposição para estudar essa proposição “neoeugenista” em diálogo com as instituições às quais Piza Jr. pertencia. Essa correspondência evidenciou que os eugenistas mexicanos não abandonaram nos anos 1960 a campanha eugênica, mesmo após a reconfiguração global da eugenia pós-1945. O documento também expôs as estratégias criadas pela Sociedad Mexicana de Eugenesia para tornar a eugenia menos “dura” ou “técnica”, como uma genética aplicada, e mais “humana”, como uma filosofia “humanista”. Para tanto, era necessário que os colegas eugenistas brasileiros, representados por Piza Jr., estivessem em sintonia com seus colegas do México.

A segunda correspondência da Sociedad Mexicana de Eugenesia enviada para Piza Jr. é datada em 7 de julho 1966, com assinatura do doutor Eugenio Echeverría (Arnoux) (novo presidente) e do doutor Alfredo M. Saavedra (M.) (secretário perpétuo). Os eugenistas afirmaram enviar a carta para saudar Piza Jr., mas registraram que, apesar da semelhança dos ideais humanísticos que compartilhavam, eles não tinham recebido notícias de Piza Jr. havia muito tempo. Echeverría e Saavedra (7 jul. 1966, p.1) postularam que esperavam do professor brasileiro uma colaboração amistosa na construção da eugenia “humanista” e em favor dos princípios que a Sociedad Mexicana de Eugenesia sustentava, orientada em torno do lema “ *Por una humanidad mejor* ”.

Considerando que a sociedade foi criada em setembro de 1931, conforme consta no próprio papel timbrado da instituição, questionamo-nos sobre como e quando Piza Jr. estabeleceu o primeiro contato com os eugenistas mexicanos. Ao afirmar na carta de 1966 que não tinham recebido notícias de Piza Jr. há tempos, podemos concluir que o professor brasileiro respondeu à correspondência inicial enviada em 1960. Qual teria sido a natureza dessa resposta? Teria o professor acatado, ao longo da década de 1960, o chamado da Sociedad Mexicana de Eugenesia para reconfigurar a eugenia a partir de uma filosofia “humanista”? Teria Piza Jr. participado ativamente na reconstrução do movimento eugenista na segunda metade do século XX? Teria o professor “renovado” sua campanha eugênica a partir dessa cooperação Brasil-México? Infelizmente não encontramos em nossa pesquisa documental os originais das respostas de Piza Jr. que nos permitiriam responder a esses questionamentos.

Em maio de 1969, Echeverría e Saavedra enviaram outra correspondência a Piza Jr., convidando o professor brasileiro para o ciclo de conferências organizado em homenagem às educadoras mexicanas, que seria realizado entre os dias 12 e 16 de maio de 1969 às 19h no auditório da Dirección General de Salubridad no Distrito Federal mexicano. A programação do evento era composta por palestras de professoras especialistas em educação maternal, educação pré-escolar e educação infantil, abordando temas diversos no campo da educação (Echeverría, Saavedra, maio 1969). Na mesma carta, os membros da Sociedad Mexicana de Eugenesia enviaram a Piza Jr. um recorte do artigo “Responsabilidad procreacional o eugenica”, justificando que era necessário definir a eugenia como “ *consciencia de responsabilidad procreacional* ” frente a tantas explicações errôneas disseminadas entre o público leigo (AMSM, maio 1969, p.4). Conjuntamente a uma carta direcionada a Renato Kehl, enviada a Piza Jr. em agosto do mesmo ano (Echeverría, Saavedra, 22 ago. 1969), essa foi a última correspondência da Sociedad Mexicana de Eugenesia que encontramos na Biblioteca Salvador de Toledo Piza Júnior.

Conforme Melo (2016) , não existem dados oficiais acerca do fim da Sociedad Mexicana de Eugenesia. A última publicação conhecida do boletim *Eugenesia* data de 1956. Entretanto, o médico Alfredo Saavedra, secretário perpétuo, membro ativo da sociedade e principal promotor da eugenia no México ( López-Guazo, 2009 ), manteve publicações avulsas em outros periódicos após essa data ( Melo, 2016 ). Nossa pesquisa documental revelou que, ao lado de Esnaurrízar e Echeverría, Saavedra foi correspondente de Kehl e Piza Jr. ao longo da década de 1960. Suas cartas foram datilografadas em papel timbrado, carimbadas com o selo da sociedade e acompanhadas de panfletos de divulgação impressos com o logotipo, caracterizando correspondências e publicações oficiais, e não apenas ações isoladas de seus membros. Contrariamente ao discurso do “fim” ou “abandono” da eugenia após a Segunda Guerra Mundial, essas correspondências nos permitem concluir que a Sociedad Mexicana de Eugenesia manteve suas atividades institucionais até pelo menos 1969, visando à reconfiguração “humanista” da eugenia em escala internacional.

## Da aposentadoria compulsória ao *Cogito, ergo sum*

As cartas de 1969 correspondem ao primeiro ano de Piza Jr. como professor aposentado da Esalq. Sua aposentadoria compulsória foi publicada pelo governo do estado de São Paulo em 29 de dezembro de 1968, ocasião em que completou 70 anos de idade. Na primeira edição da *Revista de Agricultura* de 1969, Walter Radamés Accorsi (1912-2006) publicou o texto “Prof. Salvador de Toledo Piza Jr. (decano dos decanos)”. Accorsi (1969) definiu Piza Jr. como um “ilustre pedagogo”, citando sua brilhante carreira universitária, os 48 anos dedicados ao ensino, à pesquisa e à divulgação científica, sua capacidade de prender a atenção do público nas palestras e a diversidade de temas aos quais se dedicou. Qual teria sido o alcance de Piza Jr. como referência intelectual nessas palestras proferidas em instituições de ensino e clubes sociais? Existiram outros membros que partilhavam de sua concepção racialista do período pós-1945 ou mesmo de suas teses eugenistas da década de 1930?

Encontramos uma publicação sobre eugenia assinada em nome do Rotary Club de Santa Bárbara d’Oeste, clube no qual Piza Jr. era palestrante, além de ter sido sócio-fundador da unidade de Piracicaba. O texto “Felicidade do viver social: finalidade do Rotary Clube” foi publicado em 1° de fevereiro de 1981 na “Coluna do Rotary” do jornal *Edição Barbarense* . A publicação foi assinada em nome da instituição e, segundo informação ao final do texto, foi transcrita do jornal *O Comércio* de Descalvado, pequeno município do interior paulista. No primeiro parágrafo, o texto informou que o Rotary Club defendia a democracia e a “miscigenação de todas as raças, na unidade de uma civilização superior” (Felicidade..., 1 fev. 1981, p.4). No tópico “Pensamentos sobre eugenia”, o texto mencionou frases de renomados intelectuais eugenistas, como Renato Kehl, Carlos Enrique Paz Soldán (1885-1972), (Júlio) Afrânio Peixoto (1876-1947) e Alexandre Tepedino. A frase mais radical, atribuída ao professor Hélio Garcia, dizia: “A Eugenia não diz ao homem: não tens direito de ser feliz, mas sim não tem o direito de fazer desgraçados” (Felicidade...., 1 fev. 1981, p.4).

O texto do Rotary Club definiu a eugenia como uma “escola da formação de caráter” e postulou que era preciso cuidar eugenicamente das gerações porvindouras, pois, isso não ocorrendo, “viveremos num eterno círculo vicioso a perpetuar os crimes, as doenças, os roubos, as taras, as velhacarias e todos os problemas menores” (Felicidade..., 1 fev. 1981, p.4), em uma associação entre violência social e criminalidade hereditária. Ponderou não existir qualquer esperança para a humanidade caso continuássemos cruzando indivíduos de “má qualidade” e postulou que a preocupação quantitativa, e não qualitativa com o material humano estava gerando indivíduos “inferiores” destinados a cadeias, hospitais e hospícios. Citando R.C. Calderon, o texto rotariano afirmou que, se a seleção de sementes para a agricultura e a seleção de animais de raça era uma atitude sábia, “praticar a Eugenia, que é a seleção da semente humana, é prova de maior inteligência, altruísmo e patriotismo” (Felicidade..., 1 fev. 1981, p.4).

O artigo publicado na “Coluna do Rotary” revelou que, mesmo décadas após o ápice da eugenia no período entreguerras, o ideal de criação de uma humanidade “superior” a partir da seleção eugênica se manteve vivo no Brasil. Algumas teses radicais apresentadas no texto rotariano, como a proibição da reprodução dos “degenerados” e a ideia de propensão hereditária à criminalidade, inserem-se na “linha dura” do movimento eugenista da qual Piza Jr. fez parte na década de 1930. Entretanto, apesar de o esalquiano ter sido palestrante no Rotary Club de Santa Bárbara d’Oeste, unidade responsável pela “Coluna do Rotary”, não encontramos informações suficientes para atribuir a ele a autoria do texto. Além disso, podemos observar que, contrariamente à Piza Jr., o texto rotariano não condenou a mestiçagem como degeneração.

Considerando que a autoria de “Felicidade do viver social: finalidade do Rotary Clube” não seja de Piza Jr., surgem duas questões. Primeira: se o professor da Esalq não foi uma voz isolada na campanha eugênica dentro das duas unidades regionais do Rotary Club, quem seriam os demais adeptos? Segunda: qual teria sido a influência de Piza Jr. como divulgador da eugenia nesses clubes sociais e, por conseguinte, seu papel na formação de “quadros” pró-eugenia no interior paulista na segunda metade do século XX? O que sabemos é que, mesmo após a aposentadoria compulsória, Piza Jr. continuou ativo e influente não apenas entre os frequentadores dos clubes que aclamavam suas palestras, como entre os profissionais de diversas áreas. Prova disso foi sua escolha como o “Engenheiro agrônomo do ano de 1984” pela Associação dos Engenheiros Agrônomos do Estado de São Paulo, que no *Jornal do Engenheiro Agrônomo* o definiu como um professor que “lapida consciências para a agronomia nacional” (O Agrônomo..., ago.-set. 1984, p.3).

No dia 5 de agosto de 1987, Piza Jr. assinou um termo no qual registrou a doação de sua coleção de obras científicas à USP de Piracicaba, composta por 68 trabalhos de eventos, 78 teses, 146 separatas, 185 periódicos, 2.814 livros, totalizando 3.291 itens. O termo registrou que o acervo englobava áreas como biologia, genética, embriologia, fisiologia, entomologia, zoologia, filosofia, religião, história, filologia, literatura, ficção científica, dicionários gerais e enciclopédias (Piza Jr., 5 ago. 1987), sem qualquer menção às dezenas de obras sobre o tema da eugenia, incluindo livros raros de Kehl, em sua maioria com dedicatórias afetuosas ao amigo esalquiano e companheiro da cruzada eugênica no Brasil. Proposital ou não, essa omissão que também se fez presente na nota de falecimento de Domingues (Nota de falecimento, 1972) se repetiu nos textos publicados na *Revista de Agricultura* em razão da morte de Piza Jr., em 1988 (cf. Gomes, 1988 ; Malavolta, 1988 ; Lordello, 1988 ).

No texto “‘In memorian’ ao Dr. Piza”, Luiz Gonzaga (Engelberg) Lordello (1926-2002), sucessor de Piza Jr. no departamento de Zoologia após a sua aposentadoria, informou a morte do professor, ocorrida na madrugada do dia 22 de janeiro de 1988. Destacamos duas informações publicadas na homenagem de Lordello (1988) : a dedicação de Piza Jr. na divulgação da ciência entre o público leigo e a sua longa trajetória no evolucionismo. Ambas nos ajudam a elucidar possíveis rupturas e permanências em sua campanha eugênica por meio de palestras, cursos e artigos publicados em jornais regionais. Encontramos no acervo de Piza Jr. um recorte de seu último texto, publicado no *Jornal de Piracicaba* . Intitulado “ *Cogito, ergo sum* ”, o artigo é datado de 6 de outubro de 1987, a última publicação antes de seu falecimento.

Partindo do *cogito* cartesiano, Piza Jr. (6 out. 1987) postulou que o homem foi o animal que mais e melhor desenvolveu a faculdade do pensar. Outros animais também pensavam, mas o homem já comprovou a partir de seus feitos a sua superioridade como espécie detentora da razão. O professor afirmou que nem mesmo os antepassados do homem, como o Pitecantropo, seriam capazes de pensar como o homem moderno. O Pitecantropo teria originado, segundo Piza Jr. (6 out. 1987), diferentes “tipos étnicos” que povoaram as regiões do planeta. Dentre os homens mais “civilizados”, Piza Jr. (6 out. 1987) considerou existir um grupo seleto com inteligência acima do “normal”, capaz de filosofar sobre as origens do mundo e a relação entre os seres que o povoam. Nesse processo intelectual, a mente humana criou Deus, considerado pelo professor como a suprema criação da mente universal. Assim, associando elementos da teologia cristã ao evolucionismo, tal qual fizera em sua resenha sobre o livro *Evolução* na *Revista de Agricultura* (1959-1960) e em seu Curso de Evolução (1961), Piza Jr. (6 out. 1987) encerrou sua vasta produção científica iniciada na década de 1920.

## Considerações finais

As publicações tardias de Piza Jr. nos permitem constatar que suas ações como divulgador científico se estenderam das décadas de 1940 a 1980, contexto em que renunciou à defesa pública da eugenia e reorientou sua prática a partir da vulgarização do evolucionismo darwinista, manifestamente inspirado em Haeckel. Se no início deste artigo afirmamos não ser factível delinear um quadro monolítico sobre os fundamentos epistemológicos de um intelectual com uma obra profusa, multitemática e que dialogou com os referenciais específicos de cada campo, os documentos analisados evidenciam que a extensa produção do esalquiano foi alicerçada sobre uma base eminentemente mendeliana, darwinista e haeckeliana. Por conseguinte, os três quadros emoldurados expostos na parede de sua biblioteca, mais do que meros ornamentos, constituem um indicativo do sustentáculo teórico no qual o professor da Esalq organizou a sua produção científica no transcorrer do século XX.

O programa do Curso de Evolução de Piza Jr. (1961) não nos oferece indícios da permanência da ideia de “repulsa biológica”, que garantiria, segundo Piza Jr. (jan.-mar. 1933), a vitória da consciência eugênica sobre os instintos irracionais que levavam à união interespecífica “repugnante” entre brancos e negros. O controle dos impulsos sexuais sugerido por Piza Jr. (1961) se referia à postura de autocontrole do homem na presença da mulher, sem remeter à questão racial. Contudo, a ideia da ciência como guia-mestre, sinônimo de verdade e razão esclarecida, capaz de atenuar o comportamento animalesco e irracional, se manteve presente em sua obra. O programa desse curso nos permite constatar que Piza Jr. não se desvencilhou totalmente de algumas concepções defendidas na década de 1930, momento em que encampou o movimento eugenista, dirigiu o maior periódico especializado no país e defendeu as teses radicais do racismo científico.

Os conteúdos e a frequência das correspondências enviadas pela Sociedad Mexicana de Eugenesia encontradas na Biblioteca Salvador de Toledo Piza Júnior revelaram que o professor da Esalq manteve ao longo dos anos 1960 um contato regular com os eugenistas Miguel López Esnaurrízar, Alfredo Saavedra e Eugenio Echeverría. Seria de fundamental importância para este campo de pesquisa a localização das respostas de Piza Jr. aos intelectuais mexicanos, sobretudo para confirmarmos se o esalquiano acatou ou não o convite para reconfiguração da eugenia como uma filosofia “humanista”, de modo a garantir a continuidade da campanha pelo melhoramento racial enfraquecida após a Segunda Guerra Mundial.

O breve texto “ *Cogito, ergo sum* ” denotou que, assim como no discurso de formatura proferido na Esalq em 1957 e publicado no ano seguinte na *Revista de Agricultura* , Piza Jr. (6 out. 1987) manteve a concepção racializada pautada na existência de diferentes “raças” ou “tipos étnicos”, e que continuou examinando o desenvolvimento das nações a partir da métrica do progresso civilizatório, representada pela noção de “homem civilizado” como arquétipo de evolução. O texto revelou também que parte do arcabouço discursivo dos tempos de *Boletim de Eugenia* permaneceu na obra tardia de Piza Jr. (6 out. 1987), sobretudo a classificação da evolução humana por intermédio de um padrão arbitrário e hierarquizado de normalidade, um “tipo médio” a partir do qual os “tipos étnicos” eram classificados acima ou abaixo de acordo com seu grau de desenvolvimento.

Os documentos analisados nos permitem concluir que, na segunda metade do século XX, Piza Jr. se manteve relevante no meio intelectual brasileiro, a despeito de sua marginalização no campo da genética. Confirmaram ainda a reconfiguração da eugenia no contexto pós-1945, tal qual apontado por Stepan (2005) e discutido por Carvalho e Souza (2017) . Entretanto, nossa pesquisa documental nos possibilitou identificar apenas vestígios ou “ecos” da eugenia na longa trajetória intelectual de Piza Jr., redefinida a partir da posição de divulgador do evolucionismo entre o público leigo. Se por um lado a reconfiguração liberal e “humanista” da ciência do melhoramento racial não culminou no desaparecimento das práticas autoritárias, misóginas, classistas e racistas da eugenia na sociedade brasileira após a Segunda Guerra Mundial, por outro, mais estudos serão necessários para identificarmos e confirmarmos permanências mais significativas na vasta obra do professor da Esalq. Esperamos, por fim, que nossa pesquisa se configure como um convite a novas investigações sobre o tema.

## NOTAS

1 Editado no Rio de Janeiro pelo médico e farmacêutico Renato Ferraz Kehl (1889-1974) entre 1929 e 1931, o *Boletim de Eugenia* foi o maior periódico brasileiro especializado na ciência do melhoramento racial. A partir de 1932, em decorrência da viagem de Kehl para os institutos de eugenia da Europa, o periódico passou a ser editado na cidade de Piracicaba, sob a direção de Domingues e Piza Jr. ( Habib, 2010 ).

2 A Comissão Central Brasileira de Eugenia foi fundada em 1931 para fomentar o estudo da hereditariedade, a propaganda e o apoio a medidas eugênicas no país. Sob a presidência de Renato Kehl, sua estrutura organizacional era centralizada em dez renomados eugenistas, incluindo Domingues e Piza Jr. ( CCBE, 1931 ).

3 O conflito entre os dois pesquisadores da Esalq remete ao início da década de 1930. Em um artigo publicado na *Revista de Agricultura* , Piza Jr. (1931) afirmou que a cadeira de genética que faltava no Brasil não deveria ser ocupada por “mediocridades estrangeiras”. Em 1937, conforme Habib (2010) , a Esalq admitiu Brieger para a cadeira de genética e citologia, cerca de dois anos após Piza Jr. ter sido rejeitado para o mesmo cargo. A partir de então, Piza Jr. passou a responder por processos internos em decorrência de suas divergências com seu colega de instituição. Rechaçando Piza Jr. por se dedicar mais à filosofia da ciência do que à pesquisa experimental, Brieger não reconhecia seu colega como um geneticista ( Habib, 2010 ), entendimento compartilhado por outros intelectuais no campo da genética (Araújo, Martins, 2008).

4 A associação entre ciência e cristianismo não se restringiu à obra tardia de Piza Jr., remetendo ao período em que o esalquiano foi colaborador e diretor do *Boletim de Eugenia* . Diversos textos publicados no periódico denotam o interesse de seus três diretores em tornar aceitáveis, em um país de maioria católica, as teses mais incisivas da eugenia, sobretudo o controle matrimonial e a intervenção no processo reprodutivo. Identificamos na Biblioteca Salvador de Toledo Piza Júnior duas correspondências nas quais Kehl (3 mar. 1954, 21 jul. 1960) enalteceu Piza Jr. como um intelectual capaz de persuadir o público católico, denotando que essa preocupação se manteve mesmo após o fim do periódico. Sobre as tentativas de aproximação entre eugenia e catolicismo, ver Wegner, Souza (2013).

5 Sobre a falsificação das imagens dos embriões por Haeckel, ver Hopwood (2015) .

6 Trata-se da teoria do plastinema, proposta por Piza Jr. na monografia *Localização dos fatores na linina nuclear como base de uma nova teoria sobre a hereditariedade* (1930), na qual o professor contestou a teoria cromossômica da herança de Thomas (Hunt) Morgan (1866-1945). Para uma análise específica sobre o tema, ver Araújo, Martins (2008).

7 Apesar de ser respeitado em vários campos científicos, com destaque para a citogenética de artrópodes, Piza Jr. foi profundamente criticado entre os geneticistas (Araújo, Martins, 2008). Essa marginalização veio à tona a partir dos atritos do esalquiano com a Sociedade Brasileira para o Progresso da Ciência (SBPC) na década de 1960. Em 1964, o professor publicou na *Revista de Agricultura* o artigo “O medo das ideias”, no qual propalou suas críticas à SBPC, que, segundo Piza Jr. (1964), rejeitou o artigo “Papel dos cromossômios na hereditariedade” sem avaliar o conteúdo, simplesmente por ser de sua autoria. Por esse motivo, Piza Jr. (1964, p.102) sugeriu a mudança de nome da SBPC para “SBIPC”: “Sociedade Brasileira para o Impedimento do Progresso da Ciência”. Em 1967, o biólogo e secretário-geral Wolfgang Bücherl (1911-1985) enviou uma correspondência afirmando que Piza Jr. fora eleito para o cargo de conselheiro da SBPC para o quadriênio 1967-1971 (Bücherl, 9 jun. 1967). Em resposta, Piza Jr. (24 jun. 1967) recusou o convite, afirmando que não poderia compor o conselho de uma sociedade cuja revista não aceitava artigos contrários à opinião de seus redatores.

8 Mantivemos a grafia da época para os termos “gen”, referente a genes, e “cromossômio”, referente a cromossomo.

9 De acordo com Melo (2016) , a Sociedad Mexicana de Eugenesia para el Mejoramiento de la Raza foi fundada em 1931 na capital do país. Aglutinando intelectuais de diversas áreas em torno do ideal de regeneração racial, objetivava estudar os fatores de “degeneração” que ameaçavam a humanidade, além de aplicar e divulgar as medidas eugênicas para o melhoramento racial. Possuía um boletim oficial intitulado *Eugenesia* , no qual publicaram intelectuais de diversos países latino-americanos, incluindo Renato Kehl. Sobre essa sociedade, ver López-Guazo (2009) , Fröde (2013) , Melo (2016) e Bautista (2018) .
