# Supplementary material for: Entre a divulgação científica e a eugenia tardia: rupturas e permanências na trajetória intelectual de Salvador de Toledo Piza Jr., 1898-1988
Source: Hist Cienc Saude Manguinhos. 2023 Jul 10;30:e2023025. [Article in Portuguese] doi: 10.1590/S0104-59702023000100025 (PMC10395613; doi:10.1590/S0104-59702023000100025)
Supplement: Translation

# Translation: Between scientific dissemination and late-stage eugenics: ruptures and continuations in the intellectual trajectory of Salvador de Toledo Piza Jr., 1898-1988

**DOI:** 10.1590/S0104-59702023000100025en

**Authors:** Guilherme Prado Roitberg

**Affiliations:** i Brazil Doctoral candidate, Graduate Program in Education/Universidade Federal de São Carlos.São Carlos – SP – Brazil guilhermeroitberg@gmail.com

**Keywords:** eugenics, Salvador de Toledo Piza Jr. (1898-1988, scientific racism, evolutionism, history of science

## Abstract

This article analyzes the ruptures from and continuations of eugenicist ideology in the work of Salvador de Toledo Piza Jr., a geneticist and professor at the Escola Superior de Agricultura “Luiz de Queiroz.” Documentary research involving articles, correspondence, and notes from this former director of the *Boletim de Eugenia* investigates the reshaping of eugenics in the post-1945 context, a time when Piza Jr. began to publicize evolutionism. While Piza Jr. stopped publicly defending eugenics in latter half of the twentieth century, he maintained his racialized notions into the 1950s, corresponded with eugenicist groups in the 1960s, and supported a hierarchical interpretation of human evolution until the late 1980s.

In an analysis of the reconfiguration of eugenics during the 1930s, Stepan (2005) postulated that the extreme racism in the United States, France, and especially Germany led to internal and external criticism which forced reassessment of the science of racial improvement on a larger scale. The Nuremberg Laws that banned marriage between Jews and Gentiles and the way these laws were interconnected with the eugenic legislation of the Nazi regime began to be publicized and debated around the world. News of the laws reached Latin America, but the region had already broadly disapproved of Nazi reproductive policy. During the 1930s, the US implemented a type of eugenicist reform that halted institutional investments and reviewed previous racist and classist positions.

Stepan (2005) showed that eugenics weakened in the late 1930s, but instead of disappearing it reemerged from its own ruins. This field of science was reshaped as the new field of human genetics, a supposedly neutral field without the ideological foundations of “classic” eugenics. In this way, eugenics became less “ideological” and more “scientific,” liberal, and progressive. Its external structure changed, but the central core comprising issues of gender, race, and class remained. This perspective depicts eugenics as a flexible science that was able to use new disguises to maintain its old commitment to manipulating sex and hereditary control. Its reconfiguration via human genetics allowed it to persist, even though today the term “eugenics” carries negative connotations ( Stepan, 2005 ).

This reshaping after the Second World War (1939-1945) was discussed by Carvalho and Souza (2017) , who demonstrated that the subject remained alive during the 1950s and 1960s through the work of geneticists who endeavored to deconstruct the negative image of eugenics and separate it from Nazi scientific racism. This article does not depict the factual history of eugenics from its scientific establishment by the English polymath Sir Francis Galton (1822-1911) to its reconfiguration, but rather starts with the invitation by Carvalho and Souza (2017) to investigate the ruptures and continuities in the history of eugenics after 1945. Through documentary research involving articles, lectures, syllabi, correspondence, and notes by the geneticist Salvador de Toledo Piza Jr. (1898-1988), who also taught at the “Luiz de Queiroz” College of Agriculture (Escola Superior de Agricultura “Luiz de Queiroz”, ESALQ) and headed the *Boletim de Eugenia* , we also analyze the silencing as well as potential “echoes” of eugenic ideology in his work.

This research concentrates on the sources found in the Salvador Toledo Piza Júnior Library, in the administrative and financial archives at ESALQ in Piracicaba, and in the collection of historical documentation at the Romi Foundation in the city of Santa Bárbara d’Oeste. As we shall see below, these two cities in the interior of São Paulo state were the epicenter of Piza Jr.’s long campaign to spread evolutionist thinking as a professor, researcher, disseminator of science, and “illustrious” active member of social clubs (most notably the Rotary Clubs of both cities). We also found small observations on Piza Jr. in the periodicals available in the Digital Archive of the Brazilian National Library, as well as articles by Piza Jr. in the *Revista de Agricultura* , which he founded, directed, and contributed to.

## Salvador de Toledo Piza Jr. and the Brazilian eugenics movement

Born in Capivari, in the state of São Paulo, Piza Jr. graduated from ESALQ in 1921 and joined its staff the following year as a temporary assistant in the Department of Animal Husbandry, and in 1931 became the chair of Overall and Special Zoology, Anatomy, and Comparative Physiology of Domesticated Animals. He spent his entire career as a teacher and researcher at the institution, from graduation to retirement. Alongside his colleague there, the professor and geneticist Octavio Domingues (1897-1972), Piza Jr. wrote for and served as the editor of the *Boletim de Eugenia* from 1932 to 1933.^1^

Piza Jr. was a more “theoretical” intellectual in discussions on Mendelian genetics, compared to Kehl and Domingues. He moved closer to the German research tradition after studying biology and zoology there at Berlin’s College of Agriculture and Veterinary Medicine, and covered various areas during his academic career, especially focusing on the topics of evolution and inheritability. Piza Jr. was a renowned academic, recognized for his dedication to science as well as to ESALQ, and stood out in the field of entomology, although he wrote on various topics in biology, genetics, agriculture, politics, theology, and eugenics. He published in widely circulating newspapers such as *O Estado de S. Paulo* and *Folha da Manhã* , and in periodicals with a smaller reach such as the *Jornal de Piracicaba* and *Diário de Piracicaba* ( Habib, 2010 ).

The first record of Piza Jr.’s eugenics campaign is an article entitled “Anotações à margem das ‘Lições de eugenia’ do Dr. Renato Kehl” [“Notes in the margins of ‘Eugenics lessons’ by Dr. Renato Kehl”] published in 1930 in the *Revista de Agricultura* . Piza Jr. (1930) praised and pointed out corrections to Kehl’s text, which were implemented in its second edition, published in 1935 (cf. Kehl, 1935 ). After the second phase of the *Boletim de Eugenia* , written and edited in Piracicaba, Piza Jr. began to sign his texts as a member of the Comissão Central Brasileira de Eugenia (Brazilian Central Eugenics Commission, CCBE),^2^publishing articles directly related to this topic in which he defended the radical notions of scientific racism. Notable are “O que pode resultar do casamento entre o branco e o preto” [“What can result from marriages between whites and Blacks”] (Piza Jr., jan.-mar. 1932), “O casamento do branco com o preto à luz da biologia” [“Marriage between Blacks and whites from the viewpoint of biology”] (Piza Jr., abr.-jun. 1932), and “A hereditariedade da cor da pele no casamento branco-preto” [“Heritability of skin color in Black-white marriages”] (Piza Jr., jul.-set. 1932, jan.-mar. 1933).

The articles Piza Jr. (jan.-mar. 1932, abr.-jun. 1932, jul.-set. 1932, jan.-mar. 1933, abr.-jun. 1933) published in the *Boletim de Eugenia* were based on Mendelian genetics to explain heritability. Thorough analysis of his texts in this periodical confirms that his understanding of eugenics more prominently followed Mendel rather than Galton, although Piza Jr. did not ignore Galton’s contributions and pioneering ideas in the scientific structuring of eugenics, as seen in an article entitled “Um programa para a eugenia” [“A program for eugenics”] he published in the final edition of the *Boletim de Eugenia* , April/June 1933 (Piza Jr., abr.-jun. 1933). Piza Jr.’s notion of eugenics (abr.-jun. 1932) was also guided by other authors who founded their own ideas on Mendel, such as the American geneticist and pioneer in research on fruitflies William Ernest Castle (1867-1962), the Swiss naturalist Arnold Lang (1855-1914), and the Swedish geneticist Nils Herman Nilsson-Ehle (1873-1949).

Alongside an engraving of Piza Jr. and his honorary doctorate from the Institute of Zoology in Berlin (1923), the Salvador de Toledo Piza Júnior Library at the “Luiz de Queiroz” College of Agriculture has three framed portraits representing his main theoretical references: the Austrian biologist Gregor (Johann) Mendel (1822-1884), the German biologist and naturalist Ernst (Heinrich Philipp August) Haeckel (1834-1919), and the English biologist and naturalist Charles (Robert) Darwin (1809-1882). Together with his wide-ranging collection of books (many written by Piza himself during his career as a professor and geneticist there), the archive contains over a thousand works published in his name. In the *Revista de Agricultura* alone he published 222 texts between 1926 (the year it was founded at the college) and 1987, just a few months prior to his death. With this in mind, from the beginning we did not think that these three famous authors could entirely constitute the epistemological underpinnings of the science produced by this intellectual who worked in various fields (ranging from theology to zoology) over his 89 years.

Although he also espoused the scientific racism shared by Kehl (1935) and disagreed with the notion of mixed-race viability defended by Domingues (1936) , Piza Jr.’s racial thinking was original and distinct from that of his colleagues at the *Boletim de Eugenia* . To Piza Jr. (jan.-mar. 1933, p.8-10) the existence of “perfectly white” as well as “perfectly Black” individuals was entirely possible; importantly, this indicates that his notion of eugenics was not based on the idea that “pure” Black people were synonymous with degradation, since this only occurred by mixing the Black and white “species.” In other words, Piza Jr. (jan.-mar. 1933) believed that a “pure” Black person could be a perfect individual from a eugenicist point of view, as could a “pure” white person.

The scientific racism of Piza Jr. (jan.-mar. 1933) was tied to the understanding that whites and Blacks belonged to different “species.” This thinking discouraged racial mixing, since it produced “mulattos,” “hybrids,” and “inferior” individuals derived from inter-species unions. In his view, the “aversion instinct” was common among animals; this repulsion ensured that different species did not interbreed, but rather saw each other as competitors in the struggle to survive. Humans had the power to tame their instincts through intellect, but only to a limited extent. To Piza Júnior (jan.-mar. 1933), this biological “aversion” existed in the human unconscious, although it was generally dormant. There was no spontaneous attraction, historical affinity, or phylogenetic approximation between the white and Black “species.” In Piza Jr.’s view (jan.-mar. 1933), both polygenically as well as monogenically, union between Blacks and whites was biologically “unnatural” and socially “repugnant” and consequently to be avoided.

According to Habib (2010) , Piza Jr. published an article entitled “Em torno da antropologia” [“Around anthropology”] in the *Jornal de Agronomia* at the Luiz de Queiroz College of Agriculture in 1938, in which he proposed that the study of the human species should follow the same rules and methodology as the zoological study of horses and monkeys, applying the same species divisions from zoology to human beings. This notion was based on his belief that there were different “species” (namely Blacks and whites) within *Homo sapiens* . Habib (2010) stresses that although the 1938 article did not have any apparent link to eugenics, it explicitly outlined the ideas Piza Jr. defended in the 1920s and 1930s. Central among these was “aversion” and attraction between the “species” outlined in the conclusion of an article entitled “A hereditariedade da cor da pele no casamento branco-preto” [“Heredity of skin color in Black-white marriages”] published in the next-to-last issue of the *Boletim de Eugenia* (Piza Jr., jan.-mar. 1933).

Habib states (2010, p.276-277) that Piza Jr. considered “the principal of aversion one of the main factors in evolution,” and criticized the concept of “species” that did not satisfactorily explain human evolution into several new “subspecies” or “branches.” In classifying interracial unions as irrational and ill-considered behavior, Piza Jr. suggested that inter-species crosses between whites and Blacks were just as unreasonable and abnormal as zoophilia, defining coexistence between the two “species” as psychotic cohabitation. In his view, intellectuals who believed in the notion of human brotherhood without a scientific foundation did not understand the problem of race; by resisting the subdivision of the genus into “species,” they denied biological fact ( Habib, 2010 ).

The notion he presents in his article “Em torno da antropologia” from the concept of the “aversion instinct” extended or supplemented the idea of “biological repulsion” that he developed in his article “A hereditariedade da cor da pele no casamento branco-preto” (jan.-mar. 1933), a central argument in Piza Jr.’s understanding of eugenics as detailed by Habib (2010) and Wegner (2017) . The article also indicated that Piza Jr. had more continuities than ruptures with regard to his racist readings and position against racial mixing, at least until the late 1930s. Now we shall look at other documents to identify traces of these continuations and ruptures throughout his intellectual path during the second half of the twentieth century.

## Piza Jr.’s evolutionist campaign in the post-1945 context

In 1944, the *Cidade de Santa Bárbara* newspaper reported on a visit to the Santa Bárbara d’Oeste Rotary Club by members of the Piracicaba chapter for a dinner meeting. The dinner also featured the presence of a caravan of Rotarians from the neighboring city of Americana. The article stated that Dr. Salvador de Toledo Piza Jr., president of the Piracicaba Rotary Club, spoke and appreciated the warm welcome, stating that the meeting was the start of a friendly relationship between the neighboring cities. The text makes no mention of eugenics, but was the first to reveal the influence of Piza Jr. on the region’s social clubs and his prestige as a professor at the University of São Paulo and president of the Piracicaba Rotary Club (Rotary Club, 17 dez. 1944).

On January 28, 1945, the same newspaper reported that the “renowned” Professor Piza Jr. was present at the chartering of the local Santa Bárbara d’Oeste chapter (A entrega..., 28 jan. 1945). In September of the same year, it published an article about a meeting held at the Santa Bárbara d’Oeste Rotary Club that discussed “the problem of education.” Various educators and businessmen from São Paulo and Minas Gerais were present, including the manufacturing entrepreneur Américo Emílio Romi (1896-1959) and Alfredo Maluf (1903-1951), another manufacturer and former mayor of Santo André (SP). The list of speakers at the meeting included Salvador de Toledo Piza Jr. (Uma brilhante..., 2 set. 1945); we were unable to find more information about which “problem of education” the session addressed.

In 1949, his name again appeared in a news column in Santa Bárbara d’Oeste’s *Jornal D’Oeste* . It stated that he was among the renowned guests invited to a dinner meeting at the local Rotary Club, and that his talk entitled “Animal Intelligence” was well-received (Rotary Club, 17 jul. 1949). On December 14, 1952, the *O Jornal do Povo* newspaper from the same city published a note mentioning that on December 16 at 8:00 p.m. Piza Jr. would hold “an important literary and scientific conference addressing an interesting topic,” but did not mention what that topic might be (Notícias locais, 14 dez. 1952, p.1). These two publications make it clear that Piza Jr. continued to speak on topics related to biology in the late 1940s and early 1950s, continuing his commitment to disseminating science among the lay public.

In the Salvador de Toledo Piza Júnior Library we found correspondence sent to Piza Jr. by the biologist and geneticist Ademar Freire-Maia (12 out. 1952). In a letter typed on the letterhead of the Universidade do Paraná School of Philosophy and dated October 12, 1952, Freire-Maia stated that the Center of Natural History Studies at his institution had recently held Evolution Week, during which various scientists discussed topics related to the origin of species. Because of the success of this event, new conferences would be held, and Piza Jr. would be invited to participate. Freire-Maia (12 out. 1952) justified the invite by praising Piza Jr.’s “exceptional culture” and his ongoing interest in matters of science in which he was a “brilliant specialist.”

On October 20, 1957, Piza Jr. sent a typewritten letter to Alberto Amaral, thanking him for a letter received on September 1. Piza Jr. (20 out. 1957) made it clear in the letter that he understood science to be an ongoing process of questions and discoveries, sharing his “philosophical spirit” that had been criticized by his colleague at the Agriculture College, the German geneticist Friedrich Gustav Brieger (1900-1985)^3^( Habib, 2010 ). Piza Jr. explained that he maintained his interest in the topic of human evolution, his main concern in the philosophy of science. He closed the letter (20 out. 1957) with fond regards to his friend Amaral, with some provocations on the topic of inheritability without mentioning eugenic ideas. On the other hand, detailed analysis of the publications by Piza Jr. after 1945 shows that even if he did not specifically mention the science of racial betterment, he did not renounce racialized interpretations of the Brazilian population.

In June 1958, the *Revista de Agricultura* published a text entitled “A participação do índio, do branco e do negro na etnia brasiliana” [“The participation of Indians, whites, and Blacks in Brazilian ethnicity”], a transcription of an address given by Piza Jr. at a graduation ceremony for agronomists at the formal hall of ESALQ on March 8, 1957. To mark the event, the professor had been chosen to give the commencement address. Piza Jr. (jun. 1958) started by praising the students for their appreciative attitude toward both the teaching staff and the institution. He thanked them for inviting him and said he would tell a brief story from his “innate lyricism,” which even the “hard” sciences could not stifle. Curiously, the topic of the story chosen by Piza Jr. (jun. 1958) was the participations of “the Indian,” the white man, and the Black man in the ethnic makeup of Brazil. The text does not state why he opted to introduce such a subject into a graduation ceremony for a group of agronomists.

In his address, Piza Jr. (jun. 1958, p.68) explained the ethnic formation of Brazil, describing the “Indian” as a “warrior,” the white man as an “invader,” and decrying the enslavement of and violence against the native population. Next, he revealed that at this time in the late 1950s (without mentioning the question of degeneration resulting from racial mixing) he still believed in the racial division of the Brazilian population, which he defended in his articles throughout the 1930s. In a reductionist interpretation built around broad stereotypes, Piza Jr. (jun. 1958, p.68) considered the mixed-race person a hybrid, neither white nor indigenous, who inherited the characteristics of each of these distinct “races:” “From an amorous embrace under the tropical sky was born a people that was neither Indian nor Portuguese, but had the strength and skill of the Portuguese and the love and purity of the sons of the forest” (Piza Jr., jun. 1958, p.68).

He described the indigenous conversion to Christianity through the work of the Jesuits, and then the arrival of the Black man, described as honorable and hardworking despite slavery and abuse. At the end of his address, Piza Jr. (jun. 1958) “poetically” described a Black woman who sexually “gave herself” to her “master” in exchange for gifts, a casual mention of sexual violence against Black women inserted into a graduation speech at one of the country’s leading universities. Rape and the violence of slavery became, according to Piza Jr. (jun. 1958), an everyday and caricatured “exchange of favors.” The final sentence of the address, transcribed in the *Revista de Agricultura* , reads as follows:

And of this clandestine love, which who knows if God blessed, is born a mysterious being, made of black and white, and without being either black or white was white and black: the mulatto, with wheat-colored skin, came to show us all that in Brazil two races met to never again be separated (Piza Jr., jun. 1958, p.68).

The text ends here, apparently incomplete. On the next page of this issue of the *Revista de Agricultura* , another text by another author begins. Is this how Piza Jr. (jun. 1958) ended his graduation address in the university ballroom? If not, how did this speech end, and why wasn’t it transcribed? What led Piza Jr., as the director of the *Revista de Agricultura* , to publish this address in a scientific journal? The address demonstrates that Piza Jr. (jun. 1958) continued to believe in the “mulatto” as a hybrid made by mixing two “pure” races of different origins. Finally, in 1958, two decades after he published his more racist texts, did Piza Jr. still consider mixed-race people to be “degenerates,” and marriages between Black and white people to be inter-species unions, “abominable,” “repugnant,” and irrational to the point of being comparable to zoophilia?

Months after his “intriguing” graduation address, Piza Jr. continued his work disseminating science with a speech entitled “A vida é uma luta: viver é lutar” [“Life is a struggle: living is fighting”] at the Santa Bárbara d’Oeste Rotary Club on July 1, 1958 (Recordando, 13 jul. 2017). We do not know the content of this speech. In 1959 and 1960, in the *Revista de Agricultura* Piza Jr. analyzed *Evolução* [ *Evolution* ], a book written in 1941 by Father Alejandro Roldán, a professor of anthropology and psychology at the Jesuit School of Philosophy in Spain. The book was translated into Portuguese in 1958 and featured notes by Father Emanuel C. Rondon do Amarante, a biology professor at the Jesuit School of Philosophy in Brazil. The series of five articles entitled “‘Evolução’, do padre Roldán, S.J.” [“‘Evolution,’ by Father Roldán, S.J.”] is essential to understand the epistemological foundations of Piza Jr.’s evolutionism, as well as the specific way he tried to associate Christian theology with Darwinism, a hallmark of his scientific dissemination activities which appeared in his publications until the late 1980s (Piza Jr., mar. 1959, jun. 1959, set. 1959, dez. 1959, mar. 1960).^4^

The professor drafted a critical essay, disagreeing with the author as well as the translator’s notes on various points but recognizing that the work might indicate that the Catholic church accepted evolution as a scientific truth. Piza Jr. (jun. 1959) stated that even though Catholic theologians were only partially evolutionist, acceptance of the scientifically proven facts of evolution represented enormous progress. Throughout his analysis, Piza Jr. (mar. 1959) described his inspiration by Haeckel, who in his opinion was the scientist that worked the hardest to popularize Darwin’s theory of evolution, publishing work accessible to the lay public and breaking down notions that had historically been implemented by religious fundamentalism. According to Piza Jr. (mar. 1959), Christianity’s main error was fighting evolutionary hypotheses without understanding that evolution only occurred because God clearly willed it.

In Piza Jr.’s view (mar. 1959), the Catholic church should have considered the perspective of the philosopher Saint Augustine (354-430), in which God created a material world that evolved according to the rules He Himself made; in other words, the laws that govern the transformation of all bodies in the universe were contained in the act of creation. According to Piza Jr. (jun. 1959, p.82), God created not species, but rather individuals that sexually combined to populate the Earth, which only then gave origin to the zoological species: “Only after Lamarck and Darwin did it become known that in the thinking of God, the entire process of evolution became established, through which the species, which the zoological system catalogs more and more, were formed and continued to form.”

Piza Jr. (jun. 1959) criticized creationists for placing dogma above science and consequently considering evolution a sin and the primate origins of man a heresy. Merging Christian theology and Darwinism, he defended an “enlightened Christianity” that, without renouncing its traditions, should wholly accept evolution as fact and replace the literal interpretation of “creation” with the biological notion of “transformation.” Piza Jr. (jun. 1959) emphatically stated that there was no place for creationism and spontaneous generation in the field of biology, explaining that instead of the spontaneous creation in the book of Genesis, the proven fact was that microscopic beings transformed into various species. Likewise, God did not create the species in their final forms, but instead made it possible for them to evolve through a slow, progressive process of transformations.

To Piza Jr. (mar. 1960), after Darwin evolution was no longer a problem for science; it was a historic fact and scientific truth, an obstacle only to unenlightened religious people. Through the archetype of Father Roldán, Piza Jr. tried to show the readers of the *Revista de Agricultura* that it was scientifically possible to reconcile the theories of evolution with a Christianity that did not close itself off to advances in science and technology (Piza Jr., set. 1959). He finished his reading of *Evolução* by recognizing the centennial of Darwin’s *Origin of the Species* (1859) and playing down creationist criticisms of Haeckel for having faked images of embryos to prove evolutionist theories in 1868,^5^in no way considering Haeckel’s error to have diminished the scientific value of his work and of Darwinism (Piza Jr., mar. 1960).

On July 13, 1961, a student named Murilo Graner sent a letter to Piza Jr. on letterhead from the ESALQ Academic Center, organized by students in the agronomy program. Signing as the director of the scientific division, Graner (13 jul. 1961) told Piza Jr. that the Scientific, Cultural, and Artistic Divisions of the Academic Center were going to hold a series of cultural and scientific seminars during the second semester, and asked him to teach a class on evolution and genetics in August or September as the developer of a “revolutionary” theory on chromosomes.^6^Graner’s letter (13 jul. 1961) shows that even though Piza Jr. was marginalized within the field of genetics,^7^his students considered him a reference in this area.

Documents we found in the Salvador de Toledo Piza Júnior Library revealed that while Piza Jr.’s “controversial” style caused him to be marginalized among geneticists, it also helped him gain leverage among the lay community in disseminating science. In his archive, Piza Jr. saved a clipping from the August 4, 1961 issue of the *Folha de Piracicaba* newspaper entitled “Detentor do Prêmio Nobel de Medicina será refutado pelo Prof. Toledo Piza” [“Winner of the Nobel Prize for Medicine will be disproven by Prof. Toledo Piza”]. The article stated that Piza Jr. participated in the 13th Meeting of the Brazilian Society for the Progress of Science in Poços de Caldas, Minas Gerais, and presented his work “Evolução do conceito de gen” [“Evolution of the concept of the gene”]. It also reported that in August Piza Jr. would present a lecture on “The Evolution of Species,” in which he would debate and disprove the opinion of Hermann Joseph Muller (1890-1967), the American geneticist and winner of the Nobel Prize for Medicine (Detentor..., 4 ago. 1961).

On August 24, 1961, the *Jornal de Piracicaba* newspaper announced the course in evolution that Piza Jr. would teach in the Piracicaba School of Pharmacy and Dentistry starting on the 29th of that month. The course was presented by the ESALQ Academic Center and the XXI de Abril Academic Center of the Faculdade de Farmácia e Odontologia de Piracicaba. The paper described the professor as an internationally renowned scientist, a brilliant speaker, and proponent of the chromosome theory of hereditary characteristics, defined as “a theory that denies the existence of the gene, the body thought to be responsible for determining and transmitting one or more characteristics, and is thought to be located on the chromosome” (Na Faculdade..., 24 ago. 1961, s.p.).

One of the final items listed in the program for Piza Jr.’s Course on Evolution, “instinct and intelligence” (Na Faculdade..., 24 ago. 1961), may indicate that even after ceasing to publish explicit support for eugenic ideas and being banished to the sidelines in his activities related to outreach for evolutionism, he still maintained his thinking based on controlling “animalistic” sexual instincts via eugenic notions. This can be seen in the document entitled “Temas do II Curso de Evolução” [“Topics in the 2nd Course on Evolution”] (Piza Jr., 1961), which we found in the Salvador de Toledo Piza Júnior Library. This was the program for a course led by Piza Jr. at the Piracicaba School of Dentistry in September and October 1961.

The typewritten document contains two pages; the first lists the entire course syllabus; on the second, under the heading “learn,” written in capital letters, Piza Jr. (1961) listed the principles to be taught or objectives to be attained during the course. After the initial information, Piza Jr. (1961) presented the topics covered in the program, and on the second page (after “learn”) included the four main lessons in the course. First, the idea of evolution in order to shed light on the enigmas of the universe and of life; second, evolution as a pathway to find God; third, the concept of evolution as the creation of body and soul, both of which depend on the animal body, with the spirit subordinated to its biological nature; and finally, the need for self-knowledge in studying evolution, namely knowing oneself through evolutionary foundations (Piza Jr., 1961).

Piza Jr. outlined the educational goals of this course in five points which combined to show that he may have maintained the eugenic thinking he presented in his 1933 article “A hereditariedade da cor da pele no casamento branco-preto (conclusão)” [“Heredity of skin color in Black/white marriage (conclusion)”], and indirectly in his 1938 article “Em torno da antropologia.” To Piza Jr. (1961), man could not forget his evolutionary origins and could not be a monkey; in other words, he needed to behave like the species at the top of the evolutionary ladder, using reason to guide his actions. These first two teachings were further explored in his third concept, in which man, as the bearer of reason, could not let the instinctive behavior of the monkey dominate. In the fourth item, Piza Jr. (1961) suggested that man should dominate his sexual forces and contain his “bestial instincts” when in contact with women, training himself so that Evolution (as he wrote it) would guide him to the path of truth.

We do not intend to base our hypothesis on non-historic analysis, stating that Piza Jr.’s ideas remained unchanged 31 years after he published “Anotações à margem dos ‘Lições de eugenia’ do Dr. Renato Kehl” [“Annotations on the margins of Dr. Renato Kehl’s ‘Lessons on Eugenics‘”] in the *Revista de Agricultura* , his first article on eugenics and first contribution (1930) to the eugenics campaign in Brazil. Any analysis suggesting uninterrupted continuity without disruptions, and ignoring the different contexts in which the documents were produced, may fall victim to inaccuracy and generalizations. Still, even though the evolution course program did not explicitly defend eugenics, it allows us to state that controlling sex through reason, the foundation of Piza Jr.’s notion of eugenics, still persisted (even if only implicitly) in his intellectual trajectory.

His work to continue spreading evolutionism continued throughout the 1960s, with lectures in social clubs and teaching institutions in the interior of the state of São Paulo. On October 13, 1963, one year after Piza Jr. was recognized as an Honorary Citizen of Piracicaba (Município..., 7 set. 1962), the *Jornal d’Oeste* of Santa Bárbara published an article on the Golden Jubilee celebrations for the José Gabriel de Oliveira School Group, describing the scheduling for this event which included various activities for children, parents, and teachers, and notably a lecture by Piza Jr. on October 25 entitled “Acerca da conduta animal” [“On animal behavior”] (Grupo Escolar..., 13 out. 1963). This same lecture was mentioned in the “Recordando” column of the *Diário de Santa Bárbara d’Oeste* on November 3, 2018 and November 22, 2019 (Recordando, 3 nov. 2018, 22 nov. 2019), which presents content from the past.

## The Mexican Sociedad de Eugenesia and the “humanist” reconstitution of eugenics

While Piza Jr.’s lectures at social clubs and educational institutions and publications in the *Revista de Agricultura* do not allow us to definitively state that he continued to defend the same radical racist principles of eugenics he supported in his articles in the 1930s, there is no doubt about his involvement with eugenicist intellectuals and international institutions during the 1960s. The first correspondence we identified in the Salvador de Toledo Piza Júnior Library, sent by the Sociedad Mexicana de Eugenesia,^8^features an envelope stamped October 11, 1960. The typewritten letter, signed by Miguel López Esnaurrízar (president) and Alfredo M. Saavedra (M.) (secretary), appears to be the first sent by the Mexican eugenicists to Piza Jr.. The language is formal, and the letter shares the concerns and objectives of the Mexican Sociedad de Eugenesia (Esnaurrízar, Saavedra, 11 out. 1960).

Esnaurrízar and Saavedra (11 out. 1960, p.1) stated that the motto of the Sociedad Mexicana de Eugenesia was “for a better humanity,” a phrase repeated in other correspondence and always featured in capital letters. The authors state that the society captured the essence of the philosophical trains of thought of that time, opposing the dominant thinking among scientific institutions that did not stress the “typically human” characteristics of eugenics. They believed that man should be distinguished from the animal kingdom, in a prominent position consistent with human dignity. The Mexicans asked Piza Jr. to collaborate in promoting this “humanist” conception of eugenics in which the expression “human species” should disappear since it belongs to the field of zoology (Esnaurrízar, Saavedra, 11 out. 1960).

They suggested disseminating this new conception of eugenics through scientific and educational organizations, including specialized educational literature for universities. Only in this way, wrote Esnaurrízar and Saavedra (11 out. 1960), could man reach his most elevated ideal. They also expressed their willingness to study this “neo-eugenicist” proposition in dialog with the institutions to which Piza Jr. belonged. This correspondence showed that the Mexican eugenicists did not abandon their eugenic campaign in the 1960s, even after the global reconfiguration of eugenics after 1945. The document also outlined the strategies developed by the Sociedade Mexicana de Eugenesia to make eugenics less “hard” or “technical,” like applied genetics, and more “human,” as a “humanist” philosophy. To do so, they needed Brazilian eugenicists (represented by Piza Jr.) to be in line with their colleagues in Mexico.

The second letter sent to Piza Jr. by the Sociedad Mexicana de Eugenesia is dated July 7, 1966, and signed by Dr. Eugenio Echeverría (Arnoux) (new president) and Dr. Alfredo M. Saavedra (M.) (perpetual secretary). They describe the letter as a greeting to Piza Jr., but note that despite the similar humanistic ideals they shared, they had not received news from him for some time. Echeverría and Saavedra (7 jul. 1966, p.1) indicated that they hoped Piza Jr. would be part of a friendly collaboration in constructing “humanist” eugenics espousing the principles supported by the Sociedad Mexicana de Eugenesia, centering around their motto, “for a better humanity.”

Considering that the society was created in September 1931 (as the institution’s letterhead states), we wonder how and when Piza Jr. first established contact with the Mexican eugenicists. From the comment in the 1966 letter that no news had been received from Piza Jr. for some time, we can conclude that he replied to the initial letter sent in 1960. What was the nature of that response? During the 1960s, did he respond to the call from the Sociedad Mexicana de Eugenesia to reshape eugenics according to a “humanist” philosophy? Was Piza Jr. an active participant in reconstructing the eugenics movement during the second half of the twentieth century? Did he “renew” his eugenicist campaign via this Brazil-Mexico cooperation? Unfortunately, we were unable to locate the original copies of Piza Jr.’s responses that would allow us to answer these questions during our documentary research.

In May 1969, Echeverría and Saavedra sent another letter to Piza Jr., inviting him to a conference series organized in honor of Mexican educators from May 12 to 16, 1969 at 7 p.m. in the auditorium of the Dirección General de Salubridad in the Mexican Federal District. The event consisted of lectures by teachers specializing in maternal, pre-school, and early childhood education, addressing various themes in this field (Echeverría, Saavedra, maio 1969). In the letter they included a clipping of an article entitled “Responsabilidad procreacional o eugenica” [“Reproductive responsibility: eugenics”], justifying that it was necessary to define eugenics as “the conscience of reproductive responsibility,” considering the many erroneous explanations that had been disseminated among the lay public (AMSM, maio 1969, p.4). Together with a letter to Renato Kehl, sent to Piza Jr. in August of the same year (Echeverría, Saavedra, 22 ago. 1969), this was the last correspondence from the Sociedad Mexicana de Eugenesia found in the Salvador de Toledo Piza Júnior Library.

According to Melo (2016) , there is no official data on the end of the Sociedad Mexicana de Eugenesia. The last known issue of its bulletin, *Eugenesia,* dates back to 1956. But the physician Alfredo Saavedra, its perpetual secretary, an active member of the society and the main promoter of eugenics in Mexico ( López-Guazo, 2009 ), continued to publish work in other periodicals after this date ( Melo, 2016 ). Our documentary research revealed that, alongside Esnaurrízar and Echeverría, Saavedra also corresponded with Kehl and Piza Jr. throughout the 1960s. His letters were typed on letterhead, stamped with the society’s seal, and accompanied by publicity pamphlets with its logo, indicating this was official correspondence and publications rather than isolated activities by its members. Contrary to the discourse that eugenics “ended” or was “abandoned” after the Second World War, this correspondence allows us to conclude that the Sociedad Mexicana de Eugenesia continued its institutional activities until at least 1969 in an attempt at a “humanist” reshaping of eugenics on an international scale.

## From compulsory retirement to *Cogito, ergo sum*

The 1969 letters correspond to Piza Jr.’s first year of retirement from ESALQ. His compulsory retirement was published in the official gazette of the São Paulo state government on December 29, 1968, when he turned 70 years old. In the first edition of the *Revista de Agricultura* in 1969, Walter Radamés Accorsi (1912-2006) published a text entitled “Prof. Salvador de Toledo Piza Jr. (decano dos decanos)” [“dean of deans”]. Accorsi (1969) defined Piza Jr. as an “illustrious teacher,” citing his brilliant university career, 48 years dedicated to teaching, research, and scientific dissemination, his ability to capture audiences’ attention in lectures, and the diversity of topics he devoted himself to. How far did Piza Jr.’s influence extend as an intellectual reference in these lectures at educational institutions and social clubs? Were there other members who shared his racialist concepts from the post-1945 period or even his eugenicist notions from the 1930s?

We found a publication on eugenics signed on behalf of the Rotary Club of Santa Bárbara d’Oeste, a club where Piza Jr. had spoken; he was also a founding member of the Piracicaba branch of this organization. The text, entitled “Felicidade do viver social: finalidade do Rotary Club” [“The joy of social living: the purpose of the Rotary Club”], was published on February 1, 1981 in the “Coluna do Rotary” of the *Edição Barbarense* newspaper. It was signed on behalf of the Rotary Club, and according to information at the end of the text, was reprinted from the *O Comércio* newspaper of Descalvado, a small town in the interior of the state of São Paulo. In the first paragraph, the text stated that the Rotary Club defended democracy and the “blending of all races in the unity of a higher civilization” (Felicidade..., 1 fev. 1981, p.4). Under the heading “Pensamentos sobre eugenia” [“Thoughts on eugenics”], the text presented quotes from renowned eugenicist intellectuals like Renato Kehl, Carlos Enrique Paz Soldán (1885-1972), (Júlio) Afrânio Peixoto (1876-1947), and Alexandre Tepedino. The most radical phrase, attributed to Professor Hélio Garcia, reads: “Eugenics does not say to man: you do not have the right to be happy, but rather that you do not have the right to create wretches” (Felicidade...., 1 fev. 1981, p.4).

The Rotary Club text defined eugenics as a “school for the formation of character,” claiming that eugenic care is required for future generations and until such care is undertaken “we will live in an eternal vicious cycle perpetuating crime, illness, robberies, hereditary anomalies, villainy, and all the lesser problems” (Felicidade..., 1 fev. 1981, p.4), associating social violence and hereditary criminality. It claimed that humanity has no hope if we continue to breed individuals of “poor quality,” suggesting that quantitative rather than qualitative concerns with human material were generating “inferior” individuals destined for jails, hospitals, and asylums. Quoting R.C. Calderon, the Rotary text stated that if selecting seeds for agriculture and purebred animals was wise, “practicing eugenics, which is the selection of human seed, is proof of greater intelligence, altruism, and patriotism” (Felicidade..., 1 fev. 1981, p.4).

The text in the Rotary column shows that even decades after the peak of eugenics during the period between the World Wars, the ideal of creating “superior” human beings via eugenic selection remained alive in Brazil. Some radical notions in this text, such as banning reproduction by “degenerates” and the notion of hereditary criminality, are part of the “hard line” eugenics movement which Piza Jr. participated in during the 1930s. But even though he spoke at the Rotary Club of Santa Bárbara d’Oeste, the club responsible for the newspaper column in question, we did not find any information indicating that he wrote this text. Furthermore, we note that the text does not condemn racial mixing as degeneration, in contrast with Piza Jr.’s beliefs.

If this column was not written by Piza Jr., two questions emerge. First, if Piza Jr. was not an isolated voice in the pro-eugenics campaign within these two regional branches of the Rotary Club, who were the other supporters? Second, what was his influence as a proponent of eugenics in these social clubs, and subsequently, what was his role in shaping pro-eugenics “frameworks” in the interior of São Paulo during the second half of the twentieth century? What we do know is that even after he was forced to retire Piza Jr. remained active and influential, not only among the club members who praised his lectures, but also among professionals from various areas. This can be seen in his selection as the 1984 “Agronomist of the Year” by the São Paulo State Association of Agronomist Engineers, who in the *Jornal do Engenheiro Agrônomo* defined him as a teacher who “refined consciences for Brazilian agronomy” (O Agrônomo..., ago.-set. 1984, p.3).

On August 5, 1987, Piza Jr. signed an agreement to donate his collection of scientific work to the University of São Paulo in Piracicaba; this comprised 68 event texts, 78 dissertations, 146 offprints, 185 periodicals, and 2,814 books, a total of 3,291 items. The agreement noted that the collection spanned areas such as biology, genetics, embryology, physiology, entomology, zoology, philosophy, religion, history, philology, literature, science fiction, general dictionaries, and encyclopedias (Piza Jr., 5 ago. 1987); there was no mention of the dozens of works on eugenics, including some rare books by Kehl, most containing affectionate dedications to his friend Piza Jr., his companion in the eugenics crusade in Brazil. Whether intended or not, this omission was also present in the “in memoriam” note by Domingues ( Nota de falecimento, 1972 ) and repeated in the texts published in the *Revista de Agricultura* reporting Piza Jr.’s death in 1988 (cf. Gomes, 1988 ; Malavolta, 1988 ; Lordello, 1988 ).

In the text “‘In memorian’ ao Dr. Piza” [“In memoriam: Dr. Piza Jr.”], Luiz Gonzaga (Engelberg) Lordello (1926-2002), who succeeded Piza Jr. in the Department of Zoology after his retirement, reported his death in the early hours of January 22, 1988. Two details stand out in Lordello’s homage (1988): Piza Jr.’s dedication to scientific dissemination among the lay public, and his long career in evolutionism. Both can help shed light on potential ruptures and continued participation in the eugenics campaign through lectures, courses, and articles published in regional newspapers. In the Piza Jr. archive we found a clipping from his last text, published in the *Jornal de Piracicaba* newspaper. Entitled “ *Cogito, ergo sum,* ” the article is dated October 6, 1987, the last publication before his death.

Starting from the Cartesian *cogito* , Piza Jr. (6 out. 1987) states that man was the animal that most and best developed the ability to think. Other animals also think, but through his accomplishments man has proven his superiority as the species capable of reason. Piza Jr. stated that not even man’s predecessors like pithecanthropes were able to think like modern man. Pithecanthropus, according to Piza Jr. (6 out. 1987), spawned different “ethnic types” that populated Earth’s various regions. Among the most “civilized” men, Piza Jr. (6 out. 1987) considered that a select group with higher-than-“normal” intelligence existed, capable of philosophizing about the origins of the world and the relationship between the beings that populate it. In this intellectual process, the human mind created God, which Piza Jr. considered the supreme creation of the universal mind. In this way, by combining elements of Christian theology with evolutionism (as he did in his review of the book *Evolução* in the *Revista de Agricultura* [1959-1960] and in his Course on Evolution [1961]), Piza Jr. (6 out. 1987) concluded his vast scientific production which commenced in the 1920s.

## Final considerations

The late publications of Piza Jr. show that his scientific dissemination activity extended from the 1940s to the 1980s, during which time he renounced his public defense of eugenics and redirected it to popularize Darwinist evolutionism, clearly inspired by Haeckel. While at the beginning of this article we stated it was not feasible to create a monolithic depiction of the epistemological foundations of an intellectual with profuse work spanning many fields and dialoging with specific references within each field, the documents we analyzed show that Piza Jr.’s extensive production was founded on eminently Mendelian, Darwinist, and Haeckelian concepts. In this way, the three framed portraits on Piza Jr.’s library wall were not just ornaments, but rather an indication of the theoretical underpinnings of his scientific production during the twentieth century.

The syllabus for Piza Jr.’s Course on Evolution (1961) does not provide any indications that he maintained the notion of “biological repulsion,” which according to Piza Jr. (jan.-mar. 1933) would ensure the victory of eugenic consciousness over the irrational instincts that led to “repugnant” interspecific unions between whites and Blacks. The control of sexual impulses suggested by Piza Jr. (1961) referred to man’s self-control in the presence of women, without referring to the question of race. But the idea of science as a master guide, a synonym for truth and enlightened reason capable of mitigating animal-like and irrational behavior, remained present in his work. The program for this course shows that Piza Jr. did not entirely disavow some of the notions he defended in the 1930s when he was part of the eugenics movement, directing the country’s largest journal specializing on this topic and defending the radical theses of scientific racism.

The contents and frequency of the correspondence from the Sociedad Mexicana de Eugenesia we found in the Salvador de Toledo Piza Júnior Library revealed that Piza Jr. maintained regular contact with the eugenicists Miguel López Esnaurrízar, Alfredo Saavedra, and Eugenio Echeverría throughout the 1960s. Finding Piza Jr.’s replies to these letters would be fundamentally important to this field of research, most notably to determine whether he accepted the call to reshape eugenics as a “humanist” philosophy in order to ensure the continuity of the campaign for racial improvement that lost speed after the Second World War.

The brief text “ *Cogito, ergo sum* ” indicated that, as in the graduation address given at the “Luiz de Queiroz” College of Agriculture in 1957 and published the following year in the *Revista de Agricultura* , Piza Jr. (6 out. 1987) maintained the racialized concept based on the existence of different “races” or “ethnic types” and continued to examine the development of nations based on the metric of civilizational progress, represented by the notion of “civilized man” as an archetype of evolution. The text also revealed that part of the discourse from the time that the *Boletim de Eugenia* was published persisted in Piza Jr.’s late work (6 out. 1987), especially the classification of human evolution through an arbitrary and hierarchical pattern of normality, an “average type” from which “ethnic types” were classified as higher or lower according to their degree of development.

The documents we analyzed allow us to conclude that during the second half of the twentieth century Piza Jr. remained relevant in the Brazilian intellectual milieu, despite his marginalization in the field of genetics. They also confirm the reshaping of eugenics within the post-1945 context, as pointed out by Stepan (2005) and discussed by Carvalho and Souza (2017) . However, our documentary research only found traces or “echoes” of eugenics in Piza Jr.’s long intellectual trajectory, redefined from the position of disseminating evolutionism among the lay public. Although the liberal and “humanist” reconstitution of the science of racial improvement did not culminate in the disappearance of authoritarian, misogynistic, classist, and racist practices of eugenics from Brazilian society after the Second World War, further studies are needed to identify and confirm more significant continuations of such thinking in Piza Jr.’s vast body of work. Finally, we hope that our research will be seen as an invitation to further research on this subject.

## NOTES

1 Edited in Rio de Janeiro by the physician and pharmacist Renato Ferraz Kehl (1889-1974) from 1929 to 1931, the *Boletim de Eugenia* was the largest Brazilian periodical specializing in the science of racial improvement. After Kehl traveled to Europe to visit eugenics institutes in 1932, the journal moved to the city of Piracicaba, under the direction of Domingues and Piza Jr. ( Habib, 2010 ).

2 The CCBE was founded in 1931 to encourage the study of heritability, propaganda, and to support eugenics-related activities in the country. With Renato Kehl as its president, its organizational structure was concentrated among ten renowned eugenicists, including Domingues and Piza Jr. ( CCBE, 1931 ).

3 The conflict between the two researchers at ESALQ began in the 1930s. In an article published in the *Revista de Agricultura* , Piza Jr. (1931) stated that the chair of genetics that was empty in Brazil should not be filled by “foreign mediocrities.” According to Habib (2010) , in 1937 ESALQ hired Brieger as the chair of Genetics and Cytology, roughly two years after Piza Jr. was rejected for the same position. From that time on, Piza Jr. began to be held responsible for his discrepancies with his colleague in internal proceedings. Brieger rejected Piza Jr. for dedicating himself more to the philosophy of science than experimental research, and did not recognize him as a geneticist ( Habib, 2010 ), a sentiment shared by other intellectuals in the field of genetics (Araújo, Martins, 2008).

4 The association between science and Christianity was not limited to Piza Jr.’s later work; it dates back to the period when he wrote for and directed the *Boletim de Eugenia* . Various texts published during this period expressed the interest by the three directors of this publication in making the most forceful texts on eugenics acceptable in a mostly Catholic country, particularly on the subject of controlling marriage and reproductive intervention. In the Salvador de Toledo Piza Júnior Library we found two pieces of correspondence in which Kehl (3 mar. 1954, 21 jul. 1960) praised Piza Jr. as an intellectual capable of persuading Catholic audiences, showing that this concern continued even after the end of this publication. On the attempts to bring together eugenics and Catholicism, see Wegner, Souza (2013).

5 On Haeckel’s fake images of embryos, see Hopwood (2015) .

6 This was Piza Jr.’s “plastinema” theory, proposed in his monograph *Localização dos fatores na linina nuclear como base de uma nova teoria sobre a hereditariedade* [ *Localization of factors in nuclear linin as the foundation of a new theory on heredity* ] (1930), in which he debated the chromosomal theory of inheritance proposed by Thomas (Hunt) Morgan (1866-1945). For a specific analysis of this topic, see Araújo, Martins (2008).

7 Although he was respected in various scientific fields, most notably the cytogenetics of arthropods, Piza Jr. was harshly criticized among geneticists (Araújo, Martins, 2008). This marginalization became public after friction between ESALQ and the Brazilian Society for the Progress of Science (SBPC) in the 1960s. In 1964, he published an article entitled “The fear of ideas” in the *Revista de Agricultura* , in which he listed his criticisms of the SBPC, which according to Piza Jr. (1964), rejected his article “Papel dos cromossômios na hereditariedade” [“The role of chromosomes in heredity”] without even assessing its content simply because he was the author. For this reason, Piza Jr. (1964, p.102) suggested changing the name of the SBPC to the “SBIPC”: “Sociedade Brasileira para o Impedimento do Progresso da Ciência” [“Brazilian Society for Impeding the Progress of Science”]. In 1967, the secretary general of this organization, the biologist Wolfgang Bücherl (1911-1985), sent a letter stating that Piza Jr. had been elected to serve as a board member for the 1967-1971 period (Bücherl, 9 jun. 1967). Piza Jr. (24 jun. 1967) responded by refusing the invite, saying he could not be part of the board of a society whose periodical did not accept articles that opposed the opinion of its editors.

8 According to Melo (2016) , the Sociedad Mexicana de Eugenesia para el Mejoramiento de la Raza was founded in 1931 in the country’s capital. By bringing together intellectuals from various areas to focus on ideal of racial regeneration, its goal was to study “degenerating” factors that threatened humanity, as well as to apply and disseminate eugenic measures for racial improvement. It had an official newsletter entitled *Eugenesia* , in which it published the work of intellectuals from several Latin American countries, including Renato Kehl. On this society, see López-Guazo (2009) , Fröde (2013) , Melo (2016) , and Bautista (2018) .
